# CircGPR137B/miR-4739/FTO feedback loop suppresses tumorigenesis and metastasis of hepatocellular carcinoma

**DOI:** 10.1186/s12943-022-01619-4

**Published:** 2022-07-20

**Authors:** Lianyong Liu, Mingjun Gu, Junhua Ma, Ying Wang, Miao Li, Hui Wang, Xin Yin, Xiangqi Li

**Affiliations:** 1 Department of Endocrinology and Metabolism, Punan Hospital, Pudong New District, Shanghai, 200125 China; 2 Department of Endocrinology and Metabolism, Gongli Hospital, Naval Medical University, 200135 Shanghai, China; 3 Department of Central Laboratory, Gongli Hospital, Naval Medical University, Shanghai, 200135 China; 4 Liver Cancer Institute & Zhong Shan Hospital, Fudan University, Shanghai, 200032 China; 5 Yuxi Biotechnology, Shanghai co., Ltd, Shanghai, 201615 China

**Keywords:** CircGPR137B, miR-4739, m6A, Demethylation, Hepatocellular carcinoma, FTO

## Abstract

**Background:**

Emerging evidence indicates that circular RNAs (circRNAs) and m^6^A RNA methylation participate in the pathogenesis and metastasis of multiple malignancies including hepatocellular carcinoma (HCC). However, it remains undocumented how circRNAs form a feedback loop with the m^6^A modification contributing to HCC.

**Methods:**

A novel hsa_circ_0017114 (circGPR137B) was identified from three pairs of primary HCC and adjacent normal tissues by circRNA expression profiling. The association of circGPR137B and miR-4739 with clinicopathological parameters and prognosis in patients with HCC was analyzed by RT-qPCR, fluorescence in situ hybridization and TCGA cohorts. The role of circGPR137B in HCC was estimated in vitro and in vivo. RT-qPCR, western blot, m^6^A dot blot, RIP, MeRIP and dual-luciferase reporter assays were used to validate the reciprocal regulation of the feedback loop among circGPR137B, miR-4739 and m^6^A demethylase FTO. Meanwhile, the expression, function and prognosis of FTO in HCC were investigated by RT-qPCR, western blot, TCGA and rescue experiments.

**Results:**

We identified a new dramatically downregulated circGPR137B in HCC tissues, and found that downregulation of circGPR137B or upregulation of miR-4739 was associated with poor prognosis in patients with HCC. Ectopic expression of circGPR137B strikingly repressed the proliferation, colony formation and invasion, whereas knockdown of circGPR137B harbored the opposite effects. Moreover, restored expression of circGPR137B inhibited tumor growth and lung metastasis in vivo. Further investigations showed that circGPR137B, co-localized with miR-4739 in the cytoplasm, acted as a sponge for miR-4739 to upregulate its target FTO, which mediated m^6^A demethylation of circGPR137B and promoted its expression. Thus, a feedback loop comprising circGPR137B/miR-4739/FTO axis was formed. FTO suppressed cell growth and indicated favorable survival in patients with HCC.

**Conclusion:**

Our results demonstrate that circGPR137B inhibits HCC tumorigenesis and metastasis through the circGPR137B/miR-4739/FTO feedback loop. This positive feedback mechanism executed by functional coupling between a circRNA sponge and an m^6^A modification event suggests a model for epigenetics.

**Supplementary Information:**

The online version contains supplementary material available at 10.1186/s12943-022-01619-4.

## Introduction

Hepatocellular carcinoma (HCC) is one of the most frequent pernicious tumors with higher incidence and the second cancer-related mortality in China [1] and the fifth mortality in the United States [2]. Despite the application of various novel treatments, HCC cases still have poor prognoses due to the malignant infiltration and metastasis [3]. The dysregulation of noncoding RNAs (ncRNAs) has been implicated in the pathogenesis of HCC [4, 5]. Therefore, the identification of candidate ncRNAs may offer new diagnostic and therapeutic targets for HCC.

As a novel subset of ncRNAs, circular RNAs (circRNAs) are formed by back-splicing and characterized by a covalently closed loop and high conservativeness and stability due to their resistance to RNase R [6]. Accumulating evidence shows that circRNAs actualize gene regulation in cancer by interacting with RNA-binding proteins [7–9], sponging miRNAs [10–12] and modulating protein translation [13–15]. For example, circ-ITCH, circPPP1R12A and circKDM4C act as potential biomarkers and monitor carcinogenesis [16–19]. CircRNAs also take part in HCC. They represent prognostic factors for HCC. Circ-CDYL can distinguish the early stages of HCC [20], and circRHOT1 and circTRIM33–12 indicate a prognosis in patients with HCC [21, 22]. Circ_100,338 and circSLC3A2 facilitate HCC metastasis [23, 24], whereas circTRIM33–12 and circ_0051443 suppress its progression [22, 25]. Moreover, circ_0001955 and circASAP1 can sponge miR-516a-5p/− 326/− 532-5p to promote HCC tumorigenesis [26, 27], whereas circ_101505 sponges miR-103 to repress its growth [28].

As one of the most common RNA modifications, N6-methyladenosine (m^6^A) has been indicated by increasing data to be associated with cancer progression including HCC [29]. The components of m^6^A methylation consist of m^6^A methyltransferases (METTL3/14/16, WTAP, KIAA1429 and RBM15/15B), demethylase (FTO and ALKBH5), and m^6^A recognition factors YTHDF1/2/3, YTHDC1/2, HNRNP protein families, eIF3 and IGF2BP1/2/3 [30]. It has been demonstrated that METTL3, YTHDF2, KIAA1429 and WTAP enhance HCC metastasis by m^6^A-dependent modifications [31–34].

Furthermore, m^6^A modification of circNSUN2 promotes colorectal liver metastasis [35] and m^6^A-modified circ-SORE sustains sorafenib resistance in HCC [36]. METTL14-mediated m^6^A modification of circORC5 suppresses gastric cancer progression [37]. However, it remains unclear how a circRNA forms a feedback loop with m^6^A modification contributing to HCC. We herein identified a novel circ_0017114 (circGPR137B), and found that low expression of circGPR137B or high expression of miR-4739 was associated with poor survival and tumor recurrence in patients with HCC. CircGPR137B curbed HCC tumorigenesis and metastasis through the circGPR137B/miR-4739/FTO feedback loop. Our findings imply that circGPR137B is a potential predictive biomarker and therapeutic target for HCC.

## Methods

### Clinical data

The HCC clinical data, as well as the expression of miRNAs, FTO and YTHDC2 were collected from The Cancer Genome Atlas (TCGA) dataset (https://genome-cancer.ucsc.edu). A tissue microarray (TMA) including 87 paired HCC tissue samples (Lot No. XT16–029) was purchased from Shanghai Outdo Biotech Company. Ten paired frozen HCC tissue samples were stored in liquid nitrogen in our laboratory. Protocols were approved by the Ethics Committee of Zhong Shan Hospital. The specimens were classified based on the TNM staging system, and diagnosed by two independent pathologists.

### CircRNA microarray analysis

Total RNA from 3 paired HCC tissues was quantified using the NanoDrop ND-1000. The sample preparation and microarray hybridization were conducted based on Arraystar’s standard protocols. CircRNA microarray analysis was performed as previously reported [10].

### Bioinformatic analysis

The specific binding of circGPR137B to miRNAs (miR-1249-5p, miR-3916, miR-6760-5p, miR-214-3p and miR-4739) was identified by using a circRNA profiling and miRbase database (http://www.mirbase.org /index.shtml) in HCC. The miR-4739 targets (FTO and YTHDC2) were identified by TargetScan7.0 (http://www.targetscan.org/vert_71/). The binding proteins of FTO or YTHDC2 were screened by starBasev3.0 (http://starbase.sysu.edu.cn/starbase2/index.php).

### Cell culture

Normal liver tissues, normal liver cell line LO2 and HCC cell lines (HcpG2, Hep3B, Huh6, Huh7 and SK-hep-1) were stored in our laboratory. They were cultured in Dulbecco’s Modified Eagle medium (DMEM) supplemented with 10% heat-inactivated fetal bovine serum (FBS), 100 U/ml of penicillin, and 100 μg/ml of streptomycin (HyClone) in a humidified atmosphere containing 5% CO_2_ at 37 °C.

### Fluorescence in situ hybridization (FISH)

The expression and cellular localization of circGPR137B or miR-4739 in HCC tissue samples were detected by FISH analysis. Digoxin-labeled probe sequence for circGPR137B (5′-GGCTTTGAAAATCACTCTGTGAACATAGCA-3′) and Biotin-labeled probe sequence for miR-4739 (5′-AGGGCCCCTCCGCTCCTCCTCCCTT-3′) were synthesized for FISH analysis. The specific description of FISH analysis was performed as previously reported [10]. The analysis software Image-pro plus 6.0 (Media Cybernetics, Rockville, MD, USA) was used to analyze immunofluorescence accumulation optical density (IOD).

### Quantitative real-time PCR (RT-qPCR)

Total RNA was extracted using TRIzol, reverse transcription was performed using M-MLV and cDNA amplification using the SYBR Green Master Mix kit (Takara, Otsu, Japan). Total RNA was isolated using a High Pure miRNA isolation kit (Roche) and RT-PCR using a TaqMan MicroRNA Reverse Transcription kit (Life Technologies). The nuclear and cytoplasmic fractions were isolated using NE-PER Nuclear and Cytoplasmic Extraction Reagents (Thermo Scientific). The primers used were listed in Supplementary Table S1.

### Western blot analysis

HCC cells were harvested and extracted using lysis buffer. Cell extracts were boiled in loading buffer, and equal amounts of cell extracts were separated on 15% SDS-PAGE gels. Separated protein bands were transferred into polyvinylidene fluoride membranes. The primary antibodies against anti-FTO (#ab126605, Abcam), anti-p21 (#ab109520, Abcam), anti-p27 (#25614–1-AP, Proteintech), anti-cleaved caspase-3 (#AF7022, Affinity), anti-cleaved caspase-9 (#AF5244, Affinity), anti-N-cadherin (#22018–1-AP, Proteintech), anti-E-cadherin (#AF0131, Affinity), anti-vimentin (#AF7013, Affinity), and anti-GAPDH (#5174, CST; AB-P-R 001, Xianzhi, Hangzhou) were diluted at a ratio of 1:1000 according to the instructions and incubated overnight at 4 °C.

### Luciferase reporter assay

HCC cell lines were seeded into 96-well plates and co-transfected with wild type (WT) or mutant (Mut) PRL-TK-pMIR-circGPR137B 3’UTR or PRL-TK-pMIR-FTO 3’UTR, and miR-4739 mimic or miR-NC (negative control). After 48 h of incubation, the firefly and Renilla luciferase activities were measured with a dual-luciferase reporter assay (Promega, Madison, WI, USA).

### Plasmid, shRNA and miRNA mimic transfection

Lentivirus mediated circGPR137B overexpression vector (pLV-circGPR137B) and empty vector pLV-circ-Luci (2A) puro (CON), plasmids pcDNA3.1-FTO, si-FTO, shRNA target sequence for circGPR137B (sh-circGPR137B: 5′-ATGTTCACAGAGTGATTTTCA-3′) and miR-4739 mimic or inhibitors were purchased from GenePharma (Shanghai, China). Negative control (NC), sh-NC, pcDNA3.1 or miR-NC was used as the control vectors. HCC cell lines were planted in 6-well plates 24 h prior to sh-circGPR137B, circGPR137B or miR-4739 transfection with 70% confluence, and then treated with Lipofectamine 2000 (Invitrogen, Carlsbad, CA, USA).

### MTT, colony formation, wound-healing, and transwell assays

MTT, colony formation, wound-healing, and transwell assays were conducted as previously reported [10].

### Actinomycin D and RNase R treatment

Transcription was prevented by the addition of 2 mg/ml Actinomycin D or DMSO (Sigma-Aldrich, St. Louis, MO, USA) as the negative control. Total RNA (2 μg) was incubated for 30 min at 37 °C with 3 U/μg of RNase R (Epicentre Technologies, Madison, WI, USA).

### RNA immunoprecipitation (RIP) and m^6^A RIP (MeRIP)

RIP assay was performed in HepG2 and Hep3B cell lines using a Magna RIP RNA-binding protein Immunoprecipitation Kit (Millipore) according to the manufacturer’s instructions. Antibodies for RIP assays against Ago2 and IgG were purchased from Abcam (ab5072, Rabbit polyclonal antibody, Cambridge, MA, USA). Anti-m^6^A antibody (A-1801-020, Epigentek) and Magna MeRIP m6A Kit (17–10,499, Millipore).were used for the MeRIP assay.

### In vivo tumorigenesis assay

BALB/c (nu/nu) nude mice (male, 6–8 week) were purchased from Shanghai SIPPR-BK Laboratory Animal Co. Ltd. (Shanghai, China). All the animals were handled according to institutional guidelines, and approved by the Animal Ethics Committee of Zhong Shan Hospital. HepG2 cells stably transfected with pLV-circGPR137B and empty vectors (pLV-circ-Luci (2A)puro，GenePharma，Shanghai, China) were labeled by luciferase. The mice were subcutaneously inoculated with 6 × 10^7^ HepG2 cells stably transfected with pLV-circGPR137B and empty vectors or Sk-hep-1 cells stably transfected with pLV-sh-circGPR137B/sh-NC. The tumor weight and size were measured every other day, and the tumor volume was calculated based on the formula: length × width^2^/2.

### Orthotopic implantation liver tumor model

To establish an orthotopic implantation liver tumor model, 6 × 10^7^ HepG2 cells stably transfected with pLV-circGPR137B and empty vectors which were labelled by luciferase were injected orthotopically into the left liver lobe of nude mice. Each mouse was injected with 100ul (6× 10^6^ cells). Each group had 5 mice. After 4 weeks, the luciferase signaling was collected for pLV-circGPR137B and control groups. When mice were sacrificed, the liver weight of the mice was encoded.

### Caudal vein pulmonary metastasis model

HepG2 cells stably transfected with circGPR137B or pcDNA3.1 were cultured in complete medium. When the cells were 70% confluent, the medium was replaced with fresh medium to remove dead and detached cells. Subsequently, 6 × 10^7^ cells were used to inject into mice via the tail vein. Each mouse was injected with 100ul (6× 10^6^ cells). Each group had 5 mice. The progression of pulmonary metastasis was investigated for 4 weeks.

### Hematoxylin and eosin (HE) staining

Mice tumor tissues were harvested, fixed in 4% paraformaldehyde, and preserved at normal atmospheric temperature. The liver tissues were sliced into 5 μm sections and stained with HE for the histological studies.

### Immunohistochemistry (IHC) analysis

IHC analysis of Ki-67 levels was conducted on paraffin slides of mouse liver and metastatic lung tumor tissues using anti-Ki-67 antibodies (Abcam, Cambridge, UK) and anti-FTO (#ab126605, Abcam). The detailed description of IHC was conducted as previously reported [5].

### Statistical analysis

Statistical analysis was executed with GraphPad Prism 7 (La Jolla, CA, USA). In brief, the values are expressed as the mean ± standard deviation (SD). Student’s test and analysis of variance were used for comparisons between groups. Kaplan–Meier analysis was used to evaluate the association of circGPR137B, miR-4739 or FTO with HCC prognosis. Pearson Correlation Analysis was used to analyze the correlation of circGPR137B with miR-4739. The categorical data were analyzed by chi-square of Fisher’s exact tests. A *P*-value of < 0.05 was considered statistically significant.

## Results

### Downregulation of circGPR137B is associated with poor prognosis in patients with HCC

**Fig. 1:**
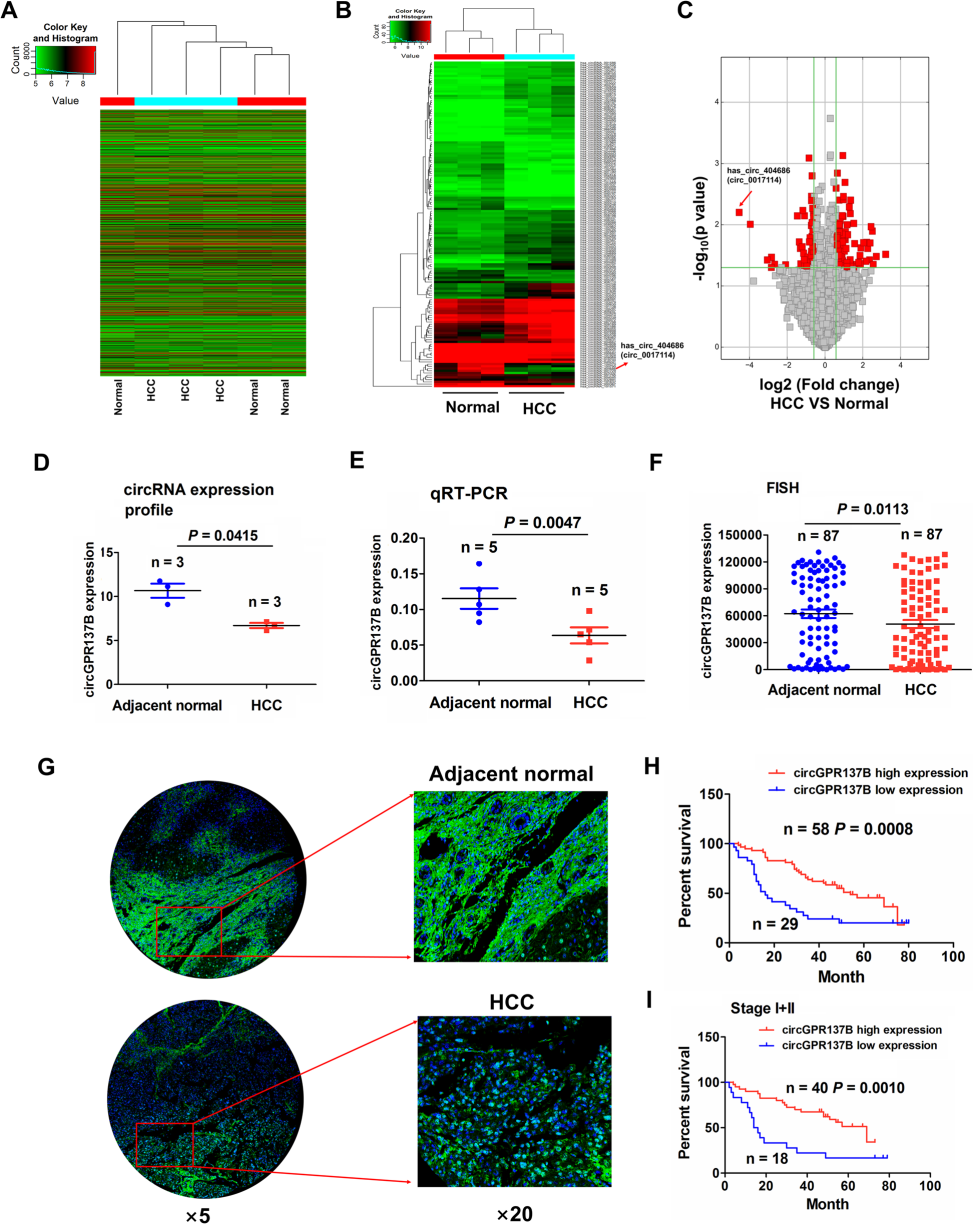
Downregulation of circGPR137B is associated with poor survival in patients with HCC. **A** CircRNA profiling analysis of the differentially-expressed circRNAs between HCC and adjacent normal tissues. **B** Heatmap analysis of 96 upregulated circRNAs and 51 downregulated circRNAs in HCC according to the restrictive conditions (*P* < 0.05 and FC > 1.5). **C** Volcano plot of a new substantially downregulated hsa_circ_0017114 (circGPR137B) in HCC tissues. **D** CircRNA profiling analysis of the expression of circGPR137B in 3 paired HCC tissues. **E** RT-qPCR analysis of the expression of circGPR137B in 5 paired HCC tissues. F, G FISH validation of the expression of circGPR137B in 87 paired HCC tissues. Blue color: DAPI; Green color: circGPR137B. **H**, **I** Kaplan–Meier analysis of the association of circGPR137B high or low expression with overall survival in HCC and early stage ones

To screen the differentially expressed circRNAs in HCC, we conducted a circRNA expression profiling in 3 paired HCC tissue samples. The heatmap indicated 6524 upregulated circRNAs and 5986 downregulated circRNAs in HCC tissues as compared with the adjacent normal tissues (Fig. 1A). According to the criteria of *P* < 0.05 and FC > 1.5, 96 upregulated circRNAs and 51 downregulated circRNAs were further identified (Fig. 1B). The volcano plot showed that hsa_circ_0017114 derived from a linear RNA GPR137B, displayed a dramatical downregulation in HCC (*P* = 0.0098; FC = 15.5613) and was nominated as circGPR137B (Fig. 1C). The circRNA profiling (Fig. 1D) and RT-qPCR (Fig. 1E) indicated a decreased circGPR137B expression in HCC as compared with the adjacent normal tissues. FISH analysis further validated this result in 87 paired HCC tissues (*P* = 0.0113; Fig. 1F, G).

According to circGPR137B expression and the prognostic data, we obtained a cutoff value of circGPR137B, and divided the cases into circGPR137B-high and circGPR137B-low groups. We found that circGPR137B harbored no relationship with the clinicopathological characteristics in HCC (Supplementary Table S2). The cases as well as the early stage ones but not the stage III cases (Supplementary Fig. S1) with circGPR137B-low expression possessed a poorer survival as compared with those with circGPR137B-high expression (Fig. 1H, I). A Cox proportional hazard model was established, and univariate and multivariate analysis unveiled that TNM stage rather than circGPR137B expression was an independent prognostic factor in HCC (Supplementary Table S3).

### The characteristics of circGPR137B in HCC cells

**Fig. 2:**
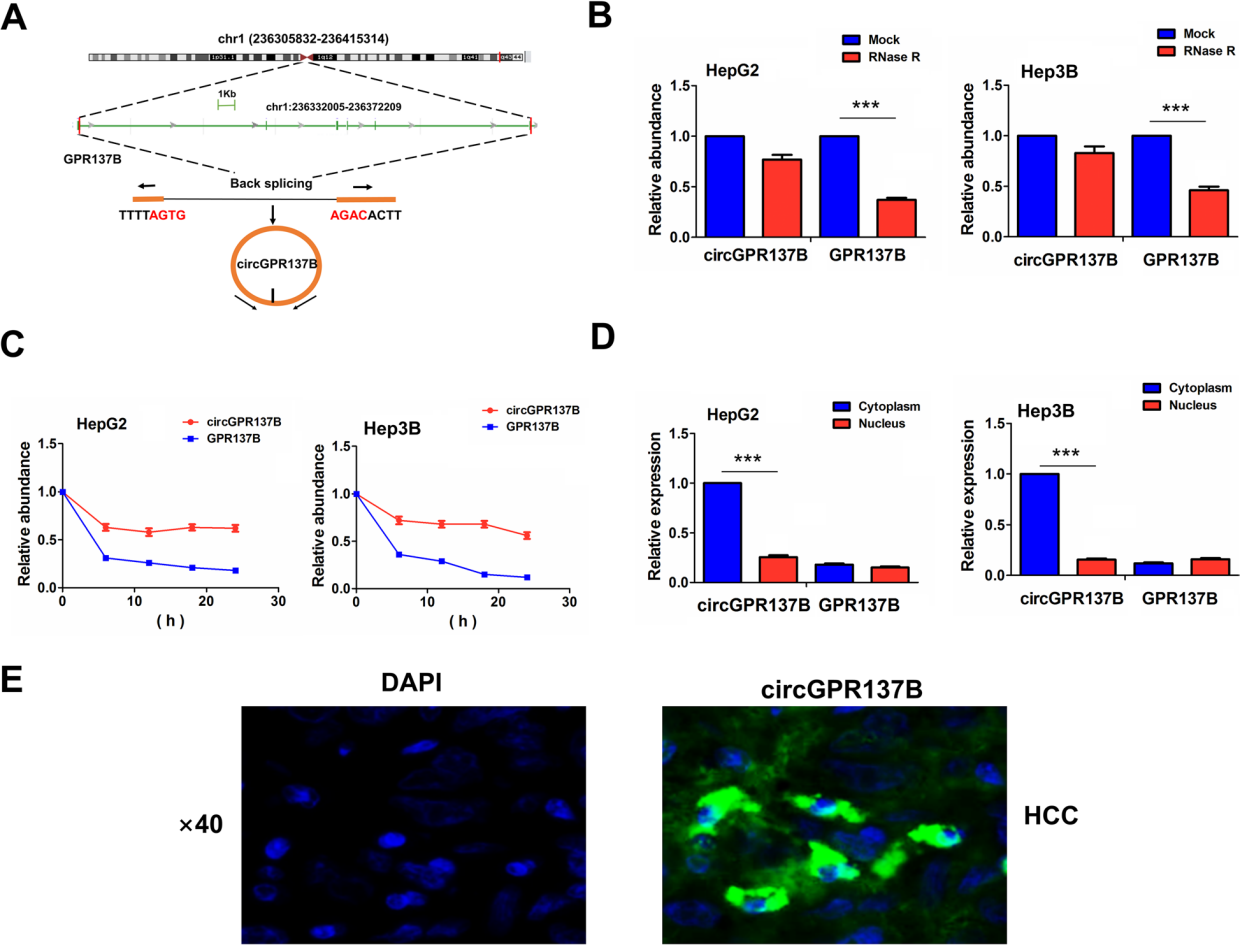
Characteristics of circGPR137B in HCC cells. **A** Genomic loci of the GPR137B and circGPR137B. **B** RT-qPCR analysis of the enrichment levels of GPR137B and circGPR137B after treatment with RNase R in HCC cell lines. **C** RT-qPCR analysis of the transcription half-life of GPR137B and circGPR137B after treatment with actinomycin D in HCC cell lines. **D**, **E** RT-qPCR and FISH analysis of the localization of circGPR137B and GPR137B in HCC cells and tissues. Blue color: DAPI; Green color: circGPR137B. Data are the means ± SEM of three experiments. ***P < 0.001

hsa_circ__0017114 (chr1:236332005–236,372,209), derived from exon 1, 7 regions within G protein-coupled receptor 137B (GPR137B) locus, is located on chromosome 14q1(q42.3), and nominated as circGPR137B, whose genomic sequence is 40,204 nt and spliced length is 1537 nt. (Fig. 2A). The mature sequence for circGPR137B is shown in supplementary data. When exposed to RNase R treatment for 2 h, GPR137B expression level was extremely lowered, but circGPR137B exhibited no significant alternation duo to its resistance to RNase R in HepG2 and Hep3B cells (Fig. 2B). We then investigated the stability of circGPR137B after exposure to the actinomycin D, an inhibitor of the transcription at indicated time points. We found that the transcription half-life of circGPR137B was much longer than that of linear GPR137B in HepG2 and Hep3B cells (Fig. 2C). The cytoplasmic or nuclear localization of circGPR137B and GPR137B was determined by qPCR and FISH analyses, which indicated that, circGPR137B was predominantly localized in the cytoplasm of HCC tissues in comparison with the linear GPR137B (Fig. 2D, E) .

### circGPR137B inhibits in vitro cell growth and invasion

**Fig. 3:**
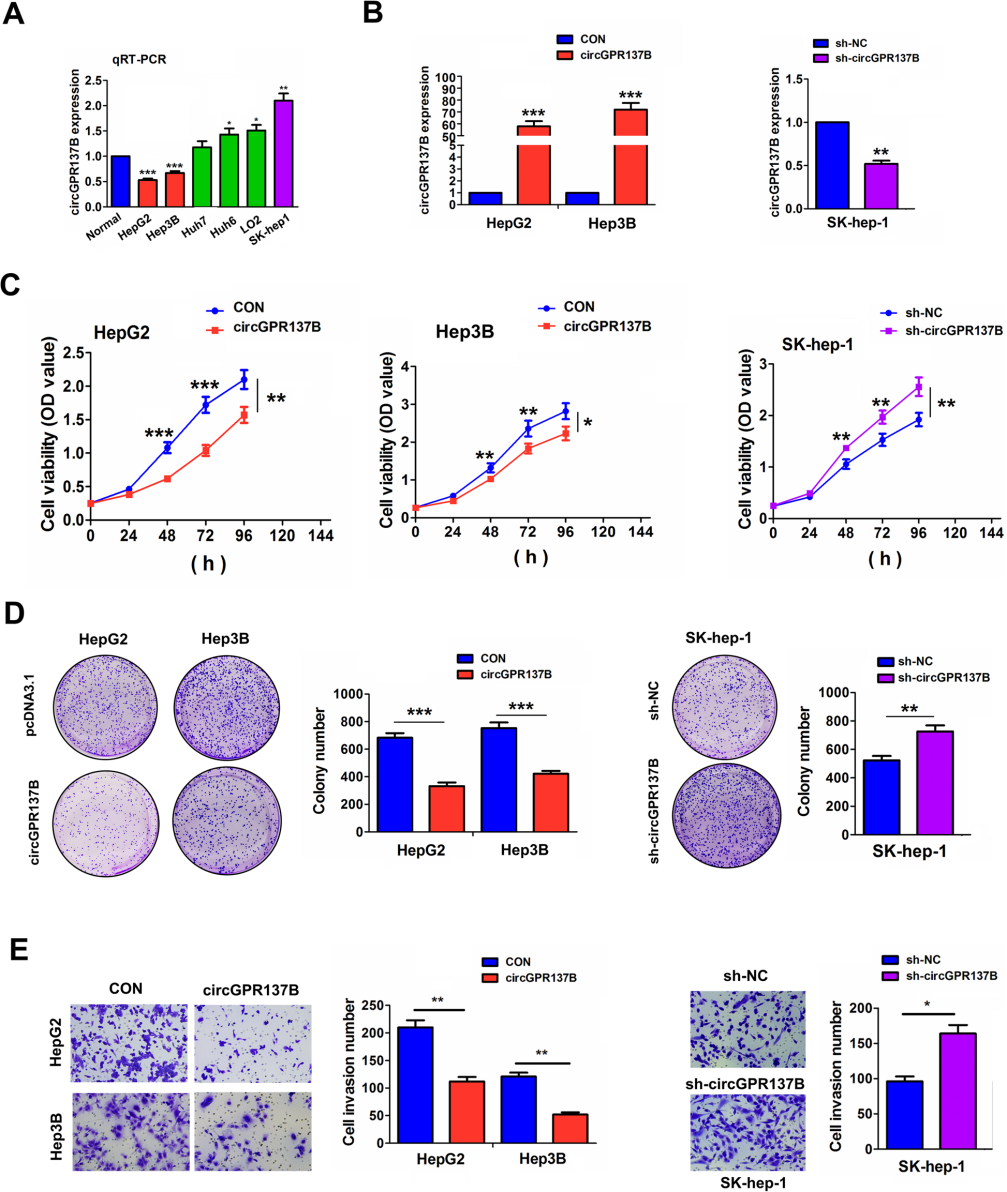
CircGPR137B inhibits in vitro cell growth. A RT-qPCR analysis of the expression levels of circGPR137B in different HCC cell lines. B RT-qPCR analysis of the overexpression efficiency of circGPR137B lentiviruses in HepG2 and Hep3B cell lines and the knockdown efficiency of sh-circGPR137B lentiviruses in SK-hep-1 cells. **C** MTT. Colony formation (**D**) and Transwell analysis (**E**) of the effects of circGPR137B overexpression or knockdown on cell proliferation, colony formation and cell invasion in HCC cell lines. Data are the means ± SEM of three experiments. *P < 0.05; **P < 0.01, ***P < 0.001

We estimated the expression of circGPR137B in multiple HCC cell lines by qRT-PCR analysis, which indicated that circGPR137B harbored a higher expression in SK-hep-1 cell line, but a lower expression in HepG2 and Hep3B cell lines in comparison with the normal liver tissues (Fig. 3A). Then, the overexpression efficiency of circGPR137B in HepG2 and Hep3B cell lines as well as the silencing efficiency of sh-circGPR137B in SK-hep-1 cell line was defined by qRT-PCR analysis (Fig. 3B). Further investigations indicated that, ectopic circGPR137B expression repressed the cell viability (Fig. 3C), colony formation (Fig. 3D) and cell invasive capabilities (Fig. 3E) in HepG2 and Hep3B cells, whereas knockdown of circGPR137B harbored the opposite effects (Fig. 3C–E).

### MiR-4739 has a negative correlation with circGPR137B expression and poor prognosis in HCC

**Fig. 4:**
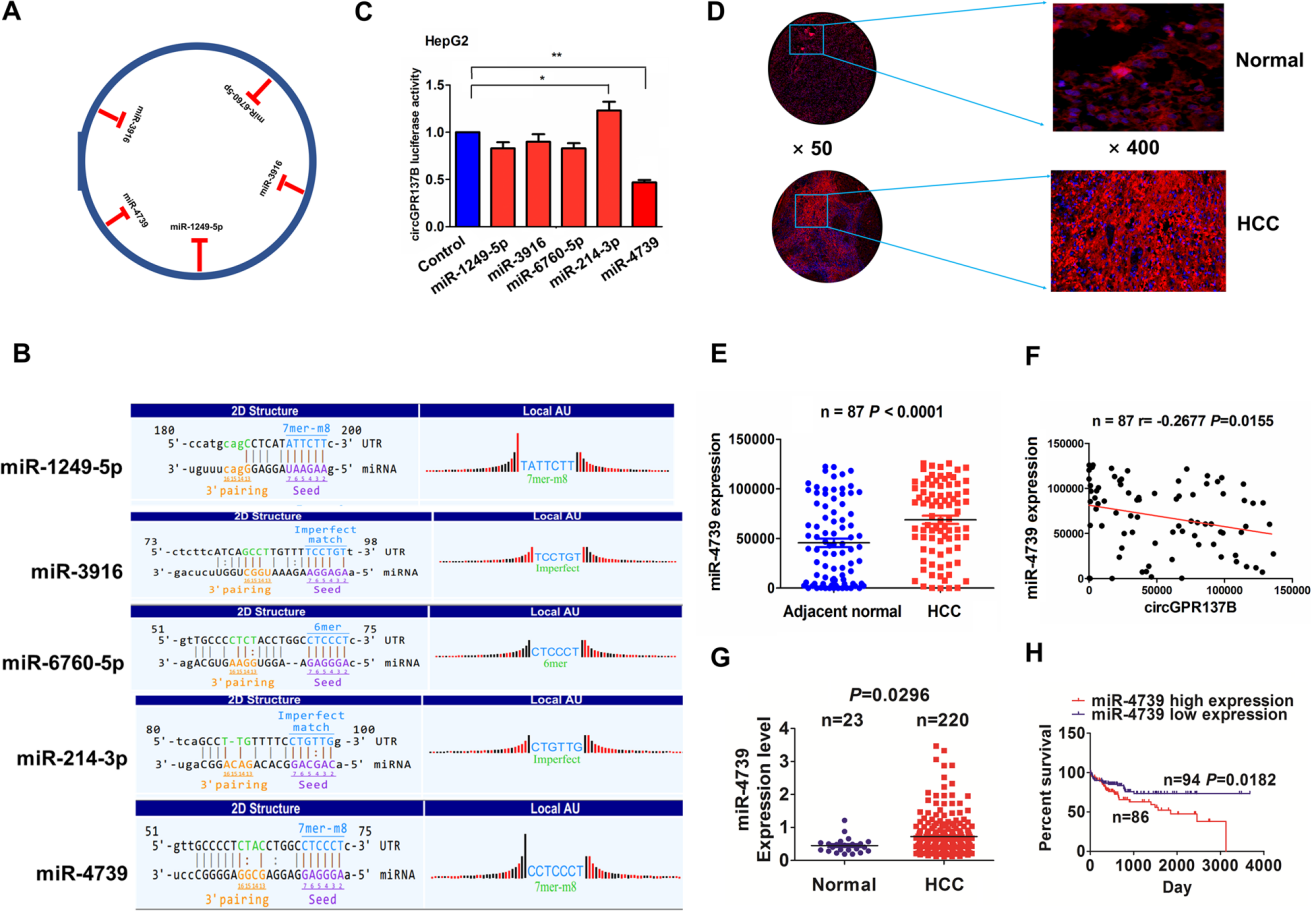
CircGPR137B has a negative correlation with miR-4739 in HCC. **A**, **B** Identification of the binding sites between circGPR137B and 5 miRNAs. **C.** Luciferase activity of circGPR137B 3’UTR was evaluated in HCC cells exposed to the treatment of 5 miRNA mimics. **D**, **E** FISH analysis of the expression levels of miR-4739 in 87 pairs of HCC tissue samples. **F** Pearson correlation analysis of the correlation of circGPR137B with miR-4739 in HCC. **G** TCGA analysis of the expression levels of miR-4739 in 220 HCC tissue samples. **H** Kaplan–Meier analysis of the association of miR-4739 high or low expression with overall survival in HCC

Depending on the circRNA expression profiling and miRbase, circGPR137B was identified to possess the potential to bind with 5 miRNAs (miR-1249-5p, miR-3916, miR-6760-5p, miR-214-3p and miR-4739) (Fig. 4A) and their binding sites can be shown in Fig. 4B. We analyzed the luciferase activity of circGPR137B in hepG2 cells after treatment with 5 miRNA mimics and found that the luciferase activities of circGPR137B were markedly reduced by miR-4739 rather than the other miRNAs (Fig. 4C). FISH analysis indicated that miR-4739 expression levels were significantly increased (Fig. 4D, E) and had a negative correlation with circGPR137B (Fig. 4F), but harbored no correlation with GPR137B in HCC (Supplementary Fig. S2). The consistent result was validated in 220 HCC tissue samples in the TCGA cohort (Fig. 4G). Then, a cutoff value of miR-4739 (0.586) was obtained and categorized the cases into miR-4739-high and miR-4739-low groups. Increased miR-4739 expression was linked to the pathological stage (*P* = 0.029) and tumor size (*P* = 0.018) in HCC (Supplementary Table S4). Kaplan–Meier analysis revealed that, patients with high miR-4739 expression harbored poorer survival as compared with those with low miR-4739 expression (Fig. 4H). The advanced cases as well as early stage ones with high miR-4739 expression harbored no differences in overall survival and tumor recurrence as compared with those with low miR-4739 expression (Supplementary Fig. S3). Univariate and multivariate analyses demonstrated that high expression of miR-4739 was an independent prognostic factor of tumor recurrence rather than poor survival in patients with HCC (Supplementary Tables S5, 6).

### CircGPR137B acts as a sponge for miR-4739 in HCC

**Fig. 5:**
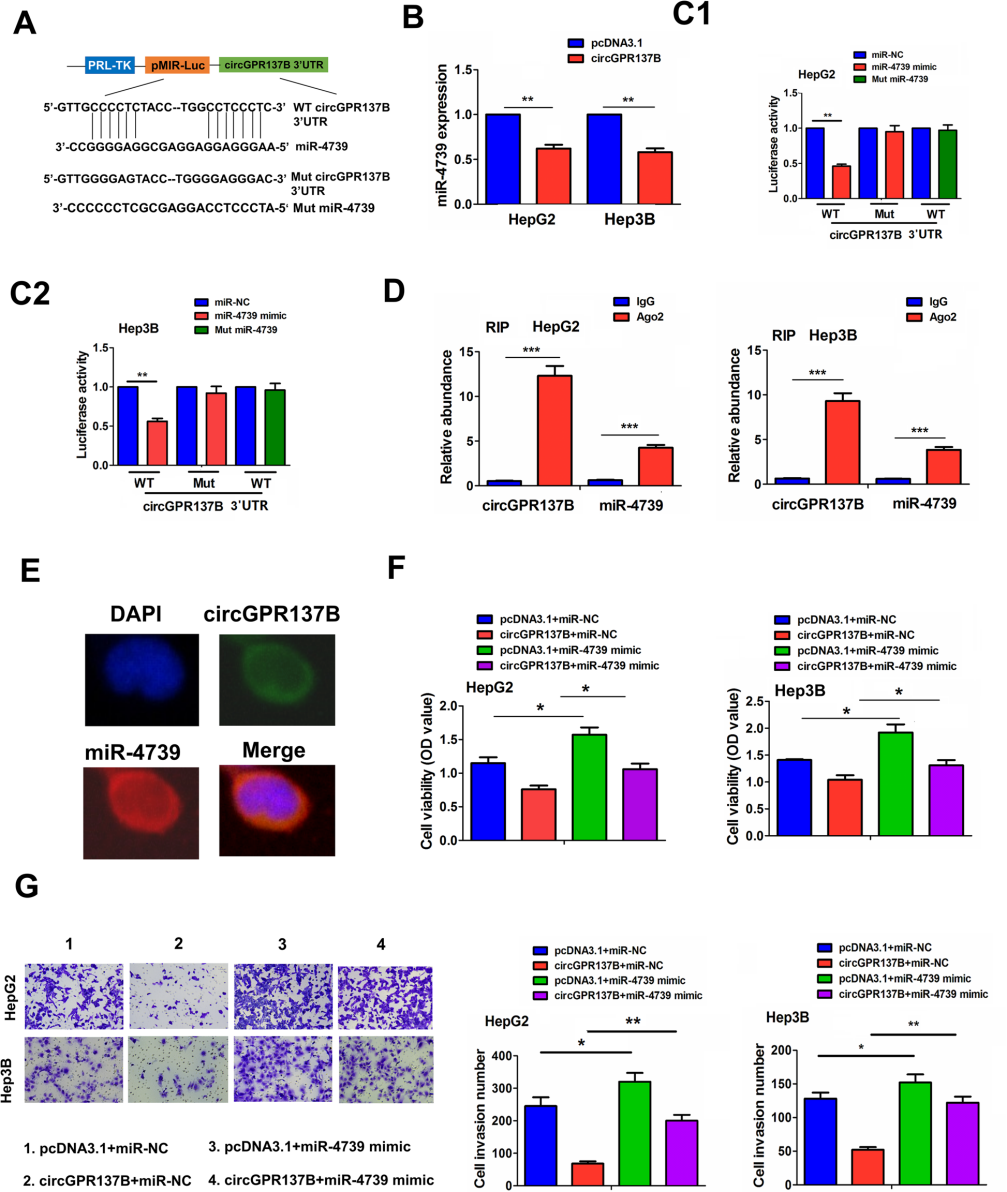
CircGPR137B acts as a sponge for miR-4739 in HCC. **A** Schematic representation of the binding sites between miR-4739 and WT circGPR137B 3’UTR. **B** RT-qPCR analysis of the effects of circGPR137B overexpression on miR-4739 expression in HepG2 and Hep3B cells. **C** Comparison of the luciferase activity of circGPR137B 3’UTR after treatment with miR-4739 mimics in HepG2 and Hep3B cells. **D.** RIP analysis of the enrichment levels of circGPR137B and miR-4739 pulled down from Ago2 or IgG protein in HepG2 and Hep3B cells. **E** FISH analysis of the co-localization of circGPR137B with miR-4739 in HepG2 cells. Blue color: DAPI, Red color: miR-4739, Green color: circGPR137B. MTT (**F**) and Transwell analysis (**G**) of the cell proliferation and invasion after transfection with circGPR137B lentiviruses and (or) miR-4739 mimics in HepG2 and Hep3B cells. Data are the means ± SEM of three experiments. *P < 0.05, **P < 0.01

The binding sites between WT circGPR137B 3‘UTR and miR-4739 can be demonstrated in Fig. 5A. The expression levels of miR-4739 were reduced by ectopic expression of circGPR137B in HepG2 and Hep3B cells (Fig. 5B), but miR-4739 mimics had no effects on circGPR137B expression (Supplementary Fig.S4). PRL-TK-pMIR-Luc vector including WT or Mut circGPR137B 3’UTR was co-transfected with miR-4739 mimics into HepG2 and Hep3B cells, and the result indicated that, miR-4739 mimics lowered the luciferase activity of WT circGPR137B 3’UTR, but exerted no effect on the luciferase activity of Mut circGPR137B 3’UTR and Mut miR-4739 also had no impact on that of WT circGPR137B 3’UTR in HepG2 and Hep3B cells (Fig. 5C). Moreover, the Ago2 occupancy in the region of circGPR137B was indicated based on an online circular RNA interactome (Supplementary Table S7). Further RIP assay implementation indicated that the enrichment of endogenous circGPR137B and miR-4739 pulled-down from Ago2-expressed HepG2 and Hep3B was markedly elevated in Ago2 pellet as compared with the input control (Fig. 5D). In addition, a cytoplasmic co-localization between circGPR137B and miR-4739 was shown in HepG2 cells by FISH analysis (Fig. 5E). The co-transfection of circGPR137B lentiviruses with miR-4739 mimics in HepG2 and Hep3B cells indicated that miR-4739 promoted the cell proliferation and invasion, and counteracted tumor suppressive effects of circGPR137B (Fig. 5F, G).

### FTO is regulated by circGPR137B/miR-4739 axis and indicates a favorable prognosis in HCC

**Fig. 6:**
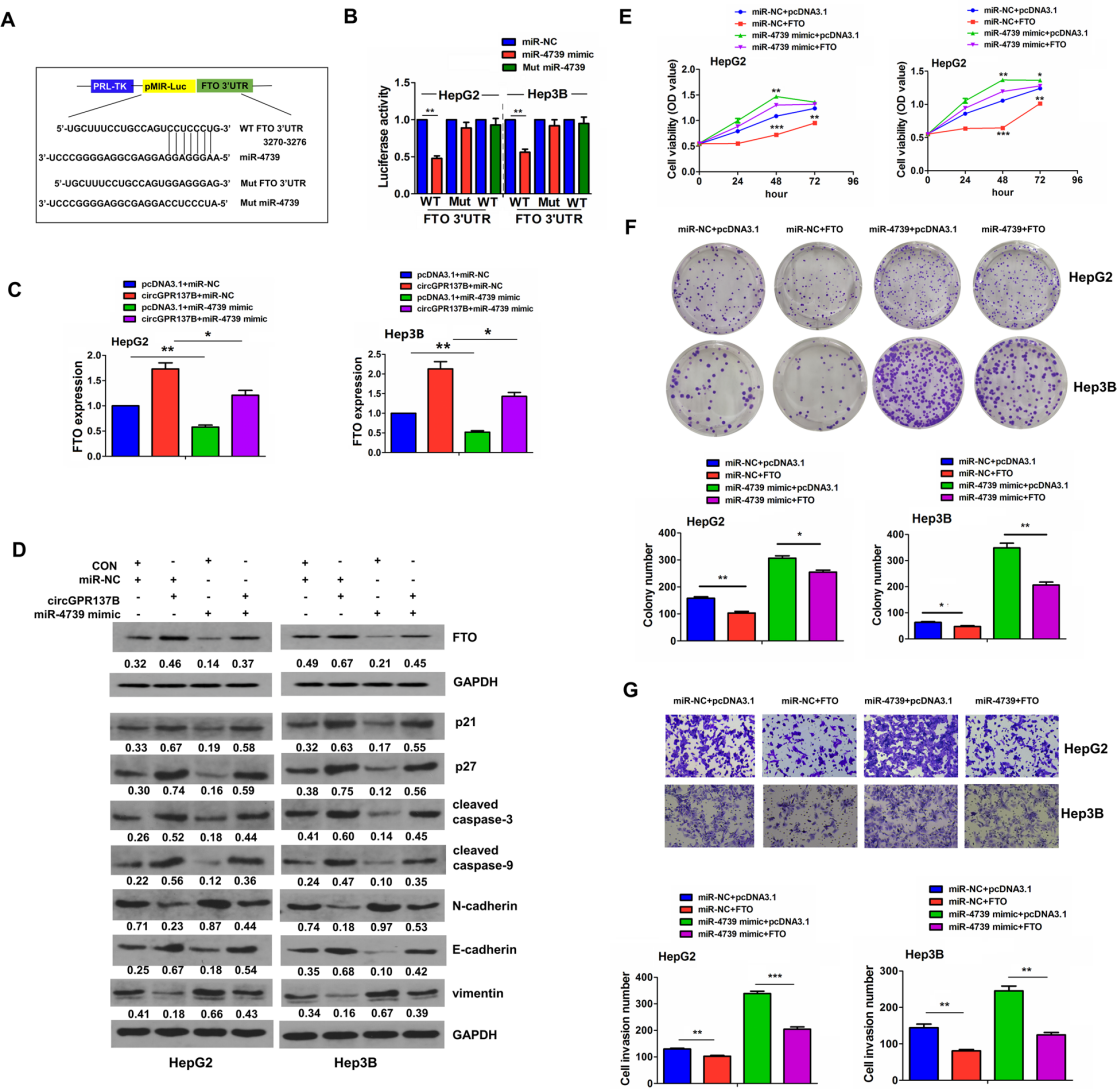
FTO is regulated by circGPR137B/miR-4739 axis in HCC. **A** Schematic representation of the binding sites between miR-4739 and FTO 3’UTR. **B** Comparison of the luciferase activity of WT or Mut FTO 3’UTR after treatment with miR-4739 mimics in HepG2 and Hep3B cells. **C**, **D** RT-qPCR and western blot analysis of the expression of FTO, p21, p27, cleaved caspase-3/− 9, N-cadherin, E-cadherin and vimentin after the transfection with circGPR137B lentiviruses and (or) miR-4739 mimics in HepG2 and Hep3B cells. **E**, **F**, **G** MTT, colony formation and transwell analysis of the cell proliferation, colony number and cell invasion after the transfection with FTO plasmids and (or) miR-4739 mimics in HepG2 and Hep3B cells. Data are the means ± SEM of three experiments. *P < 0.05, **P < 0.01, ***P < 0.001

According to the analyses of TargetScan7.1, the targets of miR-4739 were screened and m^6^A demethylase FTO might be a target of miR-4739 (Fig. 6A). It was confirmed that miR-4739 mimics could reduce the luciferase activity of WT FTO 3’UTR, but exerted no effect on Mut FTO 3’UTR and Mut miR-4739 had no impact on the luciferase activity of WT FTO 3’UTR as compared with miR-NC group in HepG2 and Hep3B cells (Fig. 6B). Furthermore, qRT-PCR (Fig. 6C) and western blot analysis (Fig. 6D) indicated that miR-4739 mimics markedly decreased expression of FTO, p21, p27, cleaved caspase-3/9 and E-cadherin and increased expression of N-cadherin and vimentin. miR-4739 also reversed circGPR137B-induced upregulation of FTO, p21, p27, cleaved caspase-3/9 and E-cadherin and downregulation of N-cadherin and vimentin in HepG2 and Hep3B cells. Then, after the co-transfection of FTO plasmids and miR-4739 mimics into HepG2 and Hep3B cells for 48 h, we found that ectopic expression of FTO prevented the cell proliferation, colony formation, and cell invasion, and reversed tumor-promoting effects caused by miR-4739 (Fig. 6E-G).

**Fig. 7:**
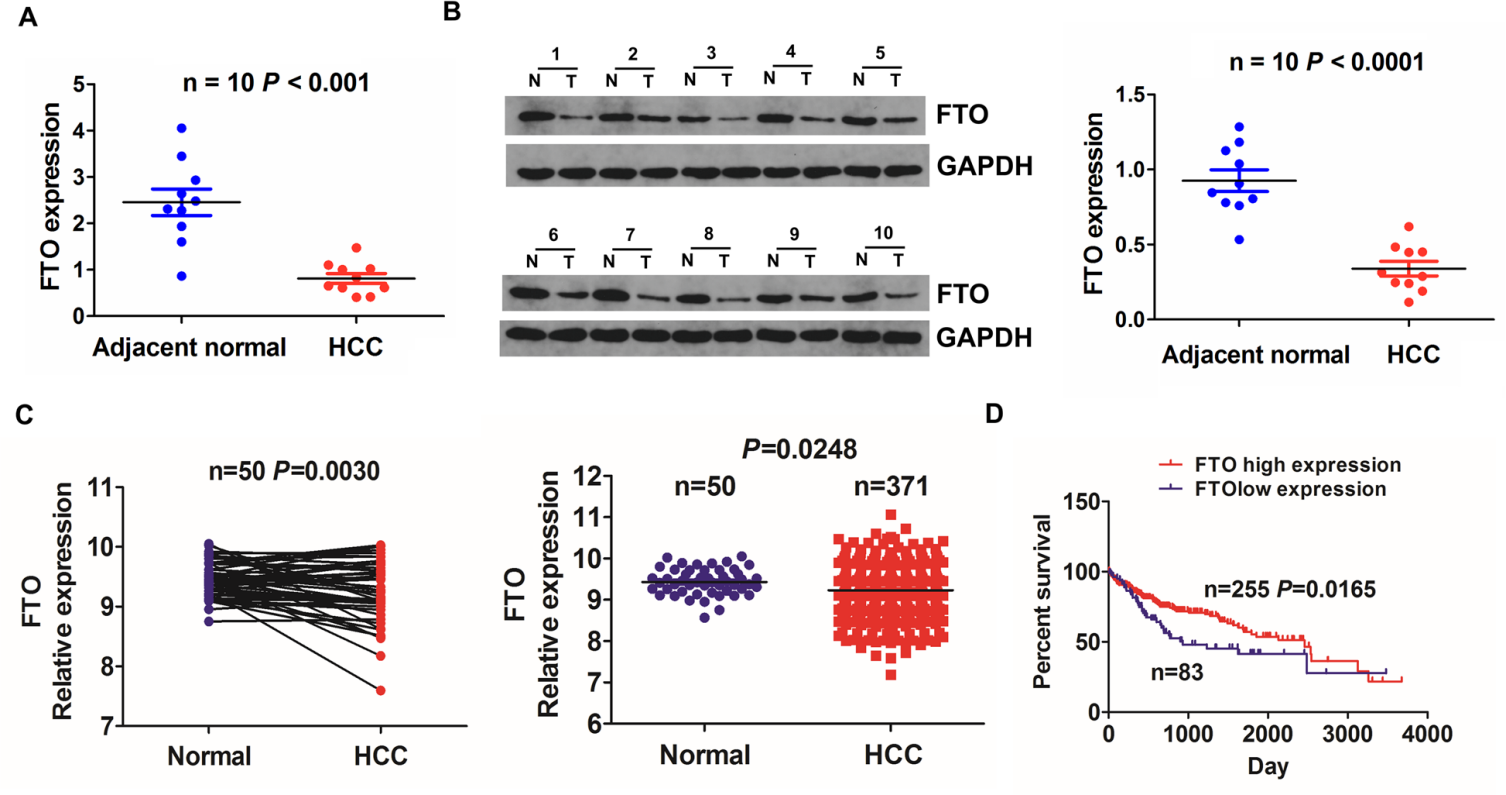
FTO downregulation indicates poor prognosis in HCC. **A**, **B** RT-qPCR and western blot analysis of the expression levels of FTO in 10 pairs of HCC samples. **C** TCGA analysis of the expression levels of FTO in 50 pairs of HCC samples and 371 cases. **D** Kaplan–Meier analysis of the association of FTO expression with overall survival in HCC

According to the RT-qPCR and western blot analyses, the expression of FTO was dramatically decreased in 10 pared HCC tissue samples as compared with the adjacent normal tissues (Fig. 7A, B). This result was validated in 50 paired and 371 unpaired HCC tissues (Fig. 7C). Then, we found that decreased expression of FTO was associated with age and distant metastasis in HCC (Supplementary Table S8). Kaplan–Meier analysis showed that the cases with high FTO expression indicated favorable survival as compared with those with low FTO expression (Fig. 7D). Univariate and multivariate analyses uncovered that FTO low expression was an independent prognostic factor of poor survival in HCC (Supplementary Table S9).

### FTO mediates m^6^A modification of circGPR137B to form a positive feedback loop with circGPR137B

**Fig. 8:**
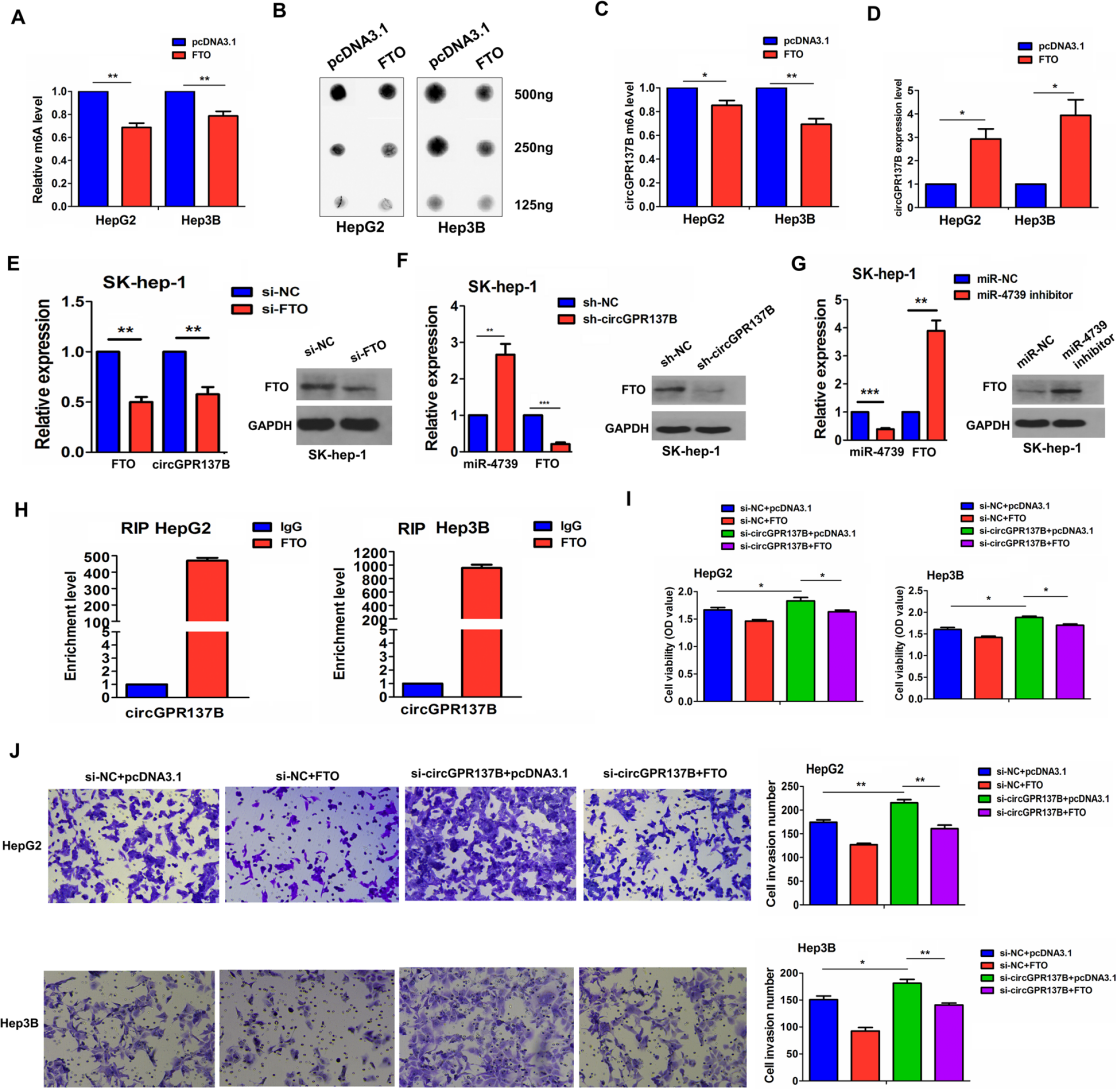
FTO mediates m^6^A modification of circGPR137B to form a positive feedback loop with circGPR137B. MeRIP (**A**) and m^6^A dot blot (**B**) analyses of the effects of FTO on the total m^6^A levels in HepG2 and Hep3B cells. **C** MeRIP analysis of the effect of FTO on the m^6^A levels of circGPR137B in HepG2 and Hep3B cells. RT-qPCR (**D**) and western blot (**E**) analysis of the effect of FTO overexpression or knockdown on the RNA levels of circGPR137B in HCC cells. RT-qPCR (**F**) and western blot (**G**) analysis of the effect of circGPR137B knockdown or miR-4739 inhibitor on FTO expression in SK-hep-1 cells. **H** RIP analysis of the binding between FTO and circGPR137B in HepG2 and Hep3B cells. MTT (**I**) and transwell (**J**) analysis of the cell proliferation and invasion after transfection with FTO and si-circGPR137B plasmids in HepG2 and Hep3B cells. Data are the means ± SEM of three experiments. *P < 0.05, **P < 0.01

It has been shown that m^6^A modification of circRNA is implicated in cancer [35–37]. We supposed that FTO-mediated m^6^A modification of circGPR137B could repress HCC progression. MeRIP and m^6^A dot blot indicated that FTO overexpression reduced the total m^6^A levels (Fig. 8A, B) and circGPR137B m^6^A levels (Fig. 8C), but increased circGPR137B RNA levels in HepG2 and Hep3B cells (Fig. 8D). Knockdown of FTO could decrease circGPR137B expression (Fig. 8E). Then, knockdown of circGPR127B increased miR-4739 expression and downregulated FTO (Fig. 8F), and the miR-4739 inhibitor increased FTO expression in the SK-hep-1 cell line (Fig. 8G). RIP assays validated that FTO could bind to circGPR137B in HCC cells (Fig. 8H). However, FTO had no effects on GPR137B m^6^A levels and could not bind to GPR137B (Supplementary Fig. S5). Further functional assays demonstrated that knockdown of circGPR137B promoted cell proliferation and invasion and counteracted FTO-induced antitumor effects in HepG2 and Hep3B cells (Fig. 8I, J).

### CircGPR137B represses in vivo HCC tumorigenesis

**Fig. 9:**
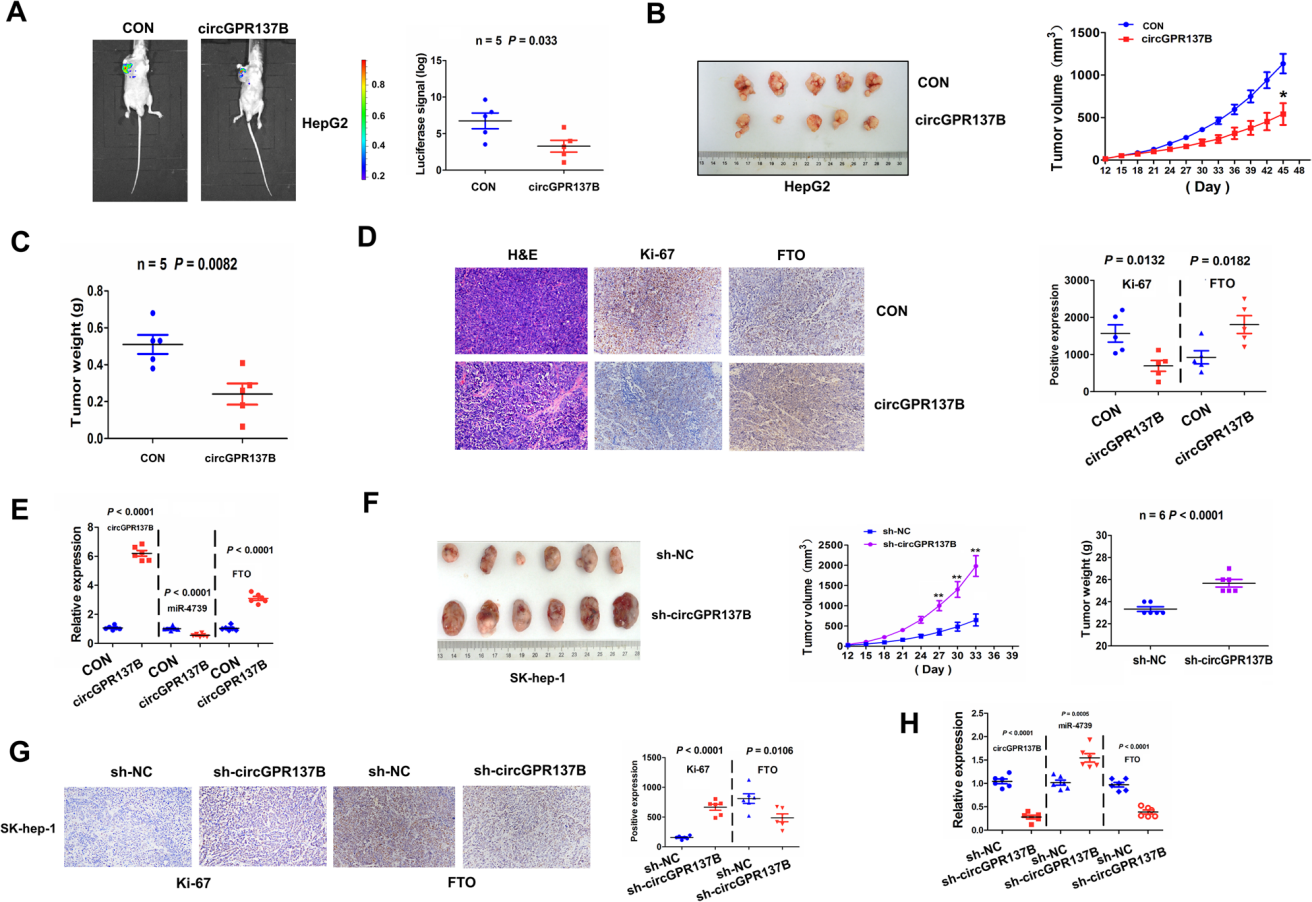
CircGPR137B inhibits in vivo HCC tumorigenesis. **A** Ex vivo imaging of the luciferase signals in tumors formed by stable transfection with circGPR137B or control group into luciferase-labeled HepG2 cells. **B**, **C** Schematic representation of the comparison of HepG2 xenograft tumors between circGPR137B and control groups and statistical analysis of the tumor size and weight between circGPR137B and control groups. **D** HE staining of the tumor tissue cells and IHC analysis of the Ki-67 proliferation index and FTO expression between circGPR137B and control groups. **E** RT-qPCR analysis of the expression levels of circGPR137B, miR-4739 and FTO between circGPR137B and control groups. **F** Schematic representation of the comparison of SK-hep-1 xenograft tumors between sh-circGPR137B and sh-NC groups, and statistical analysis of the tumor size and weight between sh-circGPR137B and sh-NC groups. **G** IHC analysis of the Ki-67 proliferation index and FTO expression levels between sh-circGPR137B and sh-NC groups. **H.** RT-qPCR analysis of the expression levels of circGPR137B, miR-4739 and FTO between sh-circGPR137B and sh-NC groups

Luciferase-labeled HepG2 cells stably transfected with circGPR137B or pcDNA3.1 were injected into the nude mice. Luciferase signals were examined using ex vivo imaging, and the result indicated that the luciferase signals of the tumor tissues in circGPR137B group were slighter than those in the control group (Fig. 9A). The tumors were removed from the mice when the mice was sacrificed, and those in the circGPR137B group seemed smaller than those in the control group. Statistical analysis indicated a decreased tumor size and weight in the circGPR137B group as compared with the control group (Fig. 9B, C). HE staining indicated a reduced tumor cell number and IHC analysis showed lowered Ki-67 and FTO levels in the circGPR137B group as compared with the control group (Fig. 9D). qRT-PCR analysis further showed that overexpression of circGPR137B decreased miR-4739 expression and increased FTO expression in the circGPR137B group as compared with the control group (Fig. 9E). Knockdown of circGPR137B in vivo exhibited a promoting effect on tumor growth (Fig. 9F). IHC analysis indicated elevated Ki-67 levels and lowered FTO levels in the sh-circGPR137B group as compared with the control group (Fig. 9G). qRT-PCR analysis further showed that knockdown of circGPR137B increased miR-4739 expression and decreased FTO expression in the sh-circGPR137B group as compared with the control group (Fig. 9H).

### CircGPR137B suppresses in vivo lung metastasis of HCC

**Fig. 10:**
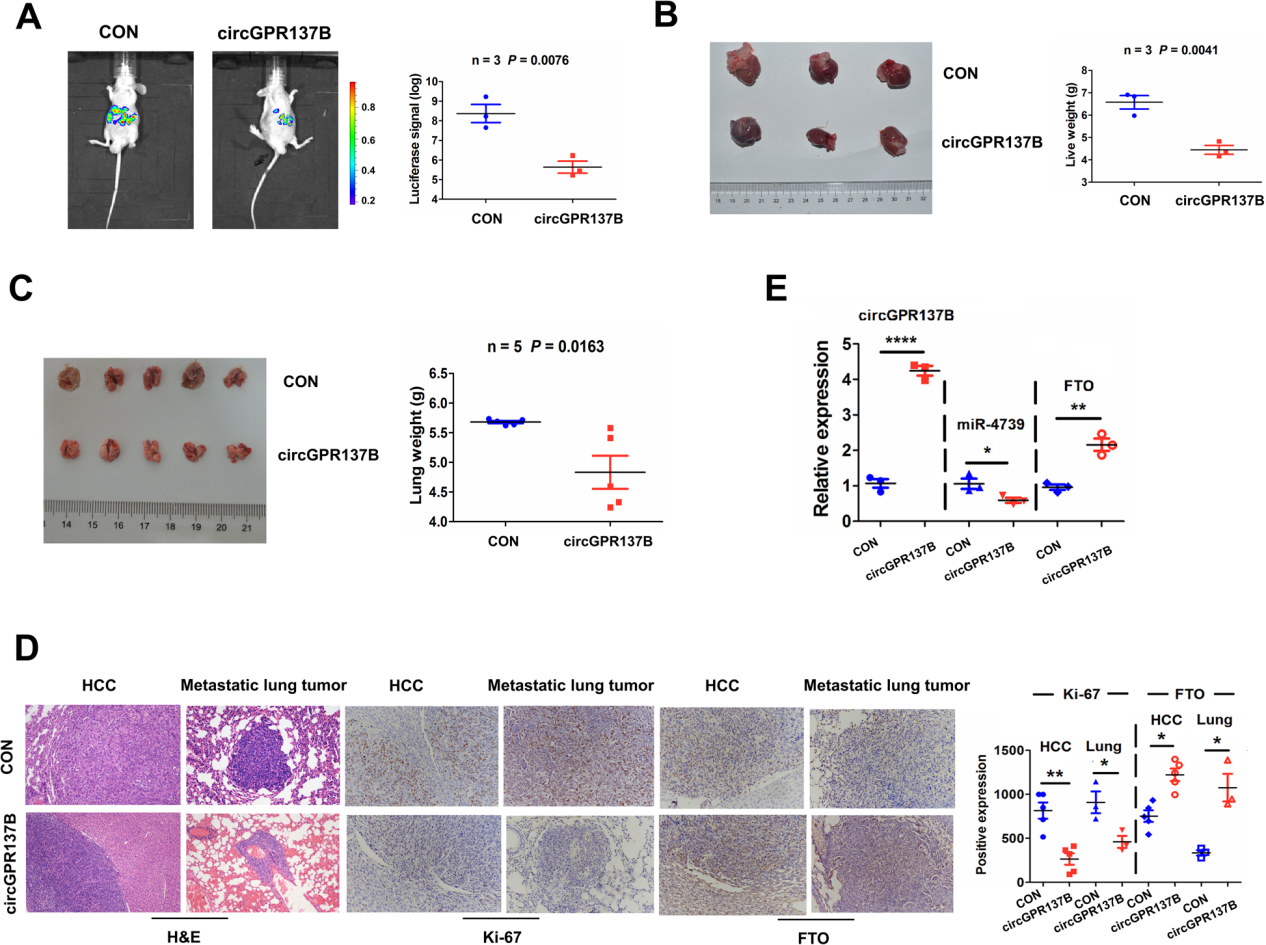
CircGPR137B inhibits in vivo lung metastasis of HCC. **A** Ex vivo imaging of the luciferase signals in orthotopic liver tumors with peritoneal infiltration by stable transfection with circGPR137B or control group into luciferase-labeled HepG2 cells. **B** Schematic representation of the comparison of orthotopic liver tumors, and statistical analysis of the total liver weight between circGPR137B and control groups. **C** Schematic representation of the comparison of metastatic pulmonary tumors, and statistical analysis of the total lung weight between circGPR137B and control groups. **D** HE staining of the tumor tissue cells from orthotopic liver tumors and metastatic pulmonary tumors in circGPR137B and control groups and IHC analysis of the Ki-67 proliferation index and FTO expression levels in orthotopic liver tumors and metastatic pulmonary tumors between circGPR137B and control groups. **E.** RT-qPCR analysis of the expression levels of circGPR137B, miR-4739 and FTO in orthotopic liver tumors between circGPR137B and control groups

Luciferase-labeled HepG2 cells stably transfected with circGPR137B or CON were injected into the mice to construct orthotopic liver tumor models. Luciferase signals were examined using ex vivo imaging, and the result demonstrated liver tumor growth with peritoneal metastasis. The luciferase signals were obviously fainter in the circGPR137B group than those in the control group (Fig. 10A). The size of the liver tumors in the circGPR137B group appeared smaller than those in the control group, and a decreased liver weight was shown in circGPR137B group as compared with the control group (Fig. 10B), but the body weight in mice had no difference between circGPR137B and control groups (Supplementary Fig. S6). Besides, HepG2 cells stably transfected with circGPR137B or CON were injected into the caudal vein to establish pulmonary metastasis models. we found that the formation rate of the metastatic lung tumors was 60.0% (3/5) in the circGPR137B group and 100.0% (5/5) in the control group, and a decreased lung weight was shown in the circGPR137B group as compared with the control group (Fig. 10C). HE staining indicated a diminished cell number in orthotopic liver tumors and metastatic lung tumor cells and IHC analysis exhibited a dwindling Ki-67 index and elevated FTO expression in liver tumors and metastatic lung tumor tissues in the circGPR137B group as compared with the control group (Fig. 10D). qRT-PCR indicated that overexpression of circGPR137B decreased miR-4739 expression and increased FTO expression in orthotopic liver tumors as compared with the control group (Fig. 10E).

## Discussion

It has been shown that upregulated circPPP1R12A [18] and downregulated circYAP1 [38] are related to the tumor metastasis and TNM stage, and can represent independent prognostic factors in patients with HCC. Herein, we identified a novel differentially-expressed circGPR137B by a circRNA profiling and validated its downregulation in HCC tissues. The downregulation of circGPR137B was associated with poor HCC survival as well as early-stage cases, and might offer a potential marker for early detection of HCC.

CircRNAs play a dual role in cancer. circPPP1R12A, circRNA-100,338 or circSLC3A2, for example, acts as an oncogenic factor [18, 23, 24], but circTRIM33–12, circ_0051443 or circYAP1 acts as a tumor suppressor in HCC [22, 25, 38]. Herein, we investigated the role of circGPR137B in HCC cells and found that circGPR137B repressed cell proliferation, colony formation, and cell invasion as well as liver tumor infiltration and pulmonary metastasis, while silencing circGPR137B harbored the opposite effects. These findings suggested that circGPR137B might be a tumor suppressor in HCC.

It is known that circRNAs can act as miRNA sponges to regulate tumor progression [26–28]. Here, we identified circGPR137B-specific binding with miR-4739 in HCC cells. MiR-4739 regulates osteogenic and adipocytic differentiation of bone marrow stromal cells [39] and pleural fibrosis [40], and act as a potential biomarker for diabetes [41]. We here found that increased miR-4739 expression was associated with pathological stage and tumor size, and acted as an independent prognostic factor of tumor recurrence in HCC. MiR-4739 can also be sponged by lncRNA VPS9D1-AS1 to prompt prostate cancer tumorigenesis [42]. We validated that circGPR137B could act as a miR-4739 sponge, and miR-4739 reversed the tumor-suppressive effects of circGPR137B, indicating that, circGPR137B might act as a sponge for miR-4739 to inhibit HCC progression.

**Fig. 11:**
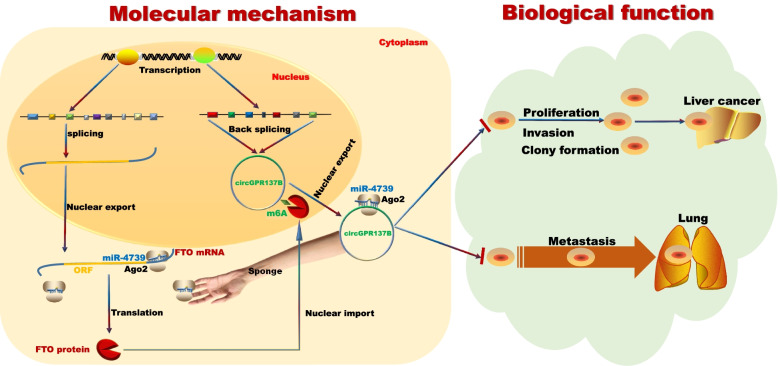
Schematic illustration of circGPR137B suppressing proliferation and metastasis of liver cancer through a positive feedback loop. CircGPR137B induces FTO protein expression by sponging miR-4739 which binds to FTO mRNA in order to inhibit its expression. FTO protein enters into cellular nuclear to mediate m6A demethylation of circGPR137B and promotes its expression. Thus, a positive feedback forms on circGPR137B production, resulting in strongly repressing liver cancer cell proliferation, colony formation, invasion, and lung migration

Increasing data show that FTO as an m^6^A demethylase plays an oncogenic role in acute myeloid leukemia [43], breast cancer [44] and melanoma [45], but restrains ovarian cancer stem cell self-renewal [46]. We here found that FTO was identified as a direct target of miR-4739 and indicated a favorable prognosis in HCC. hsa_circ_0072309 promotes tumorigenesis and invasion in non-small cell lung carcinoma by regulating miR-607/FTO axis [47]. However, our findings indicated that circGPR137B acted as a tumor suppressor in HCC by sponging miR-4739/FTO axis. Modification of circRNAs m^6^A can regulate cancer progression [35–37]. We further confirmed that FTO modulated m^6^A-dependent modification of circGPR137B and demonstrated that circGPR137B inhibited the tumorigenesis and metastasis of HCC through the circGPR137B/miR-4739/FTO feedback loop (Fig. 11).

## Conclusion

Altogether, our findings demonstrate that, downregulation of circGPR137B or upregulation of miR-4739 is associated with poor prognosis in HCC. Restored circGPR137B suppresses the malignancy of HCC through the circGPR137B/miR-4739/FTO feedback loop. Our study offers direct evidence for a feedback loop formed by circRNA and m6A modification, producing new insights into epigenetics.

## Supplementary Information

**Additional file 1: Supplementary Figure S1.** Kaplan–Meier analysis of the association of circGRP137B high or low expression with overall survival in advanced stage cases. **Supplementary Figure S2.** TCGA analysis of the expression levels of GPR137B and its correlation with miR-4739 expression in HCC tissue samples. **Supplementary Figure S3.** Kaplan–Meier analysis of the association of miR-4739 high or low expression with (A) overall survival and (B) tumor recurrence in HCC and early/late stage cases. **Supplementary Figure S4.** qPCR analysis of the effects of miR-4739 mimics on the expression of circGPR137B in HepG2 and Hep3B cell lines. **Supplementary Figure S5.** MeRIP analysis of the effects of FTO on the m6A levels of GPR137B and RIP analysis of the binding between FTO and GPR137B in HepG2 and Hep3B cell lines. **Supplementary Figure S6.** Comparison of the body weight between circGPR137B and control groups in liver tumor peritoneal metastasis models.

**Additional file 2: Table S1.** The primer sequences. **Table S2.** Correlation of circGPR137B expression with clinicopathological features of HCC patients. **Table S3.** Univariate and multivariate Cox regression analysis of the association of circGPR137B with poor survival in HCC patients. **Table S4.** The correlation of miR-4739 expression with clinicopathologic characteristics of HCC patients. **Table S5.** Cox regression analysis of miR-4739 expression as survival predictor. **Table S6.** Cox regression analysis of miR-4739 expression as recurrence predictor. **Table S7.** RNA-binding protein Ago2 sites matching flanking regions of circGPR137B. **Table S8.** The correlation of FTO expression with clinicopathologic characteristics of HCC patients. **Table S9.** Cox regression analysis of FTO expression as survival predictor.

## Data Availability

All data generated or analysed during this study are included in this published article and its additional files.

## Abbreviations

m6A: N6-methyladenosine
METTL3/14/16: methyltransferase 3/1416
ALKBH5: alkB homolog 5, RNA demethylase
GPR137B: G protein-coupled receptor 137B
FTO: fat mass and obesity-associated protein
YTHDF1/2/3: YTH N6-methyladenosine RNA binding protein 1/2/3
RIP: RNA immunoprecipitation
TCGA: The Cancer Genome Atlas
FISH: Fluorescence in situ hybridization
MiRNA: MicroRNA
HCC: Hepatocellular carcinoma
GC: Gastric cancer
CircRNA: Circular RNA
TMA: Tissue microarray
FBS: Fetal bovine serum
IOD: Immunofluorescence accumulation optical density
RT- q PCR: Quantitative real-time PCR
TNM: Tumor-Node-Metastasis
