# Supplementary figures and images for: CircGPR137B/miR-4739/FTO feedback loop suppresses tumorigenesis and metastasis of hepatocellular carcinoma
Source: Mol Cancer. 2022 Jul 20;21:149. doi: 10.1186/s12943-022-01619-4 (PMC9297645; doi:10.1186/s12943-022-01619-4)

FIG.S1

# Stage III

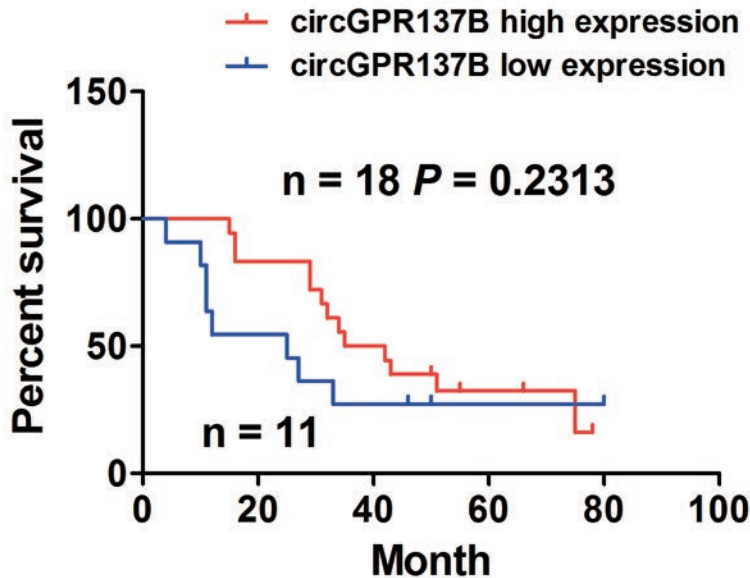

# FIG.S2

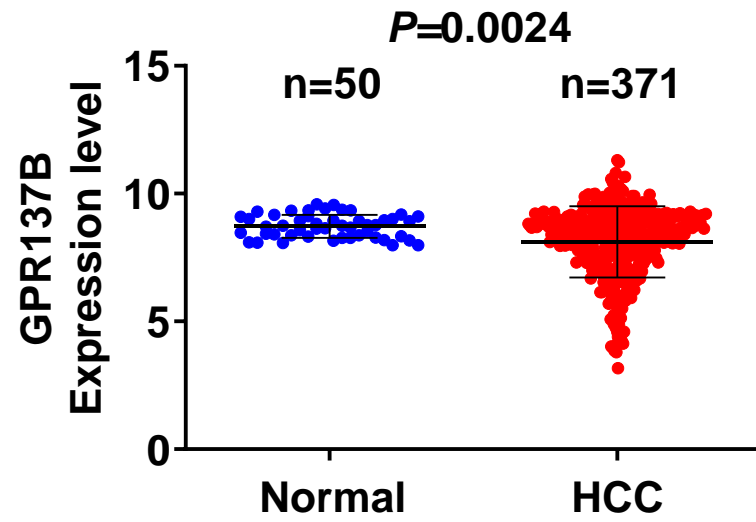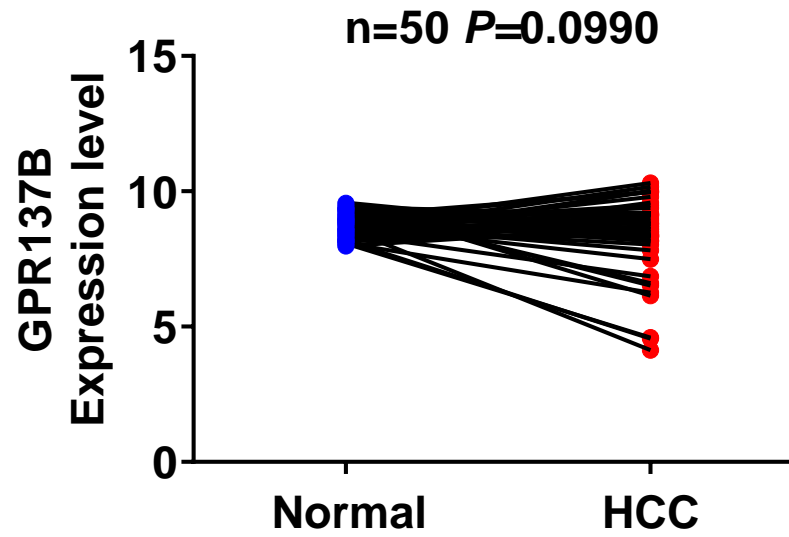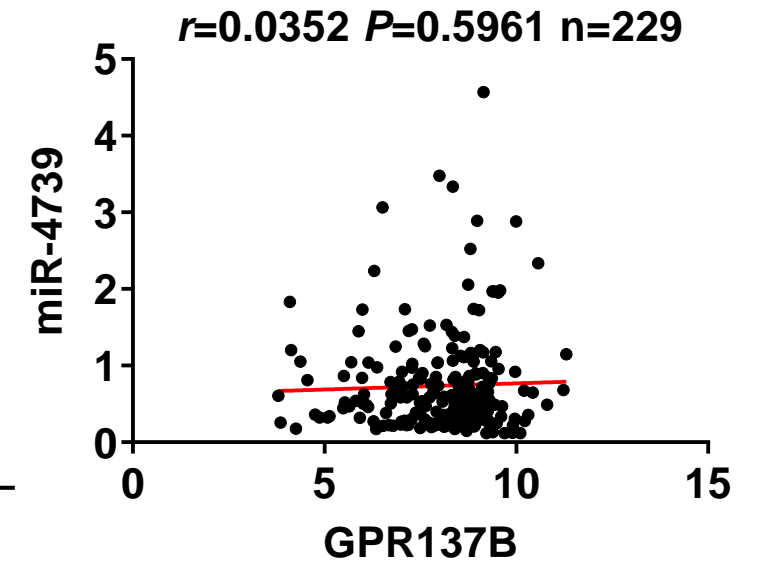

**FIG.S3****A**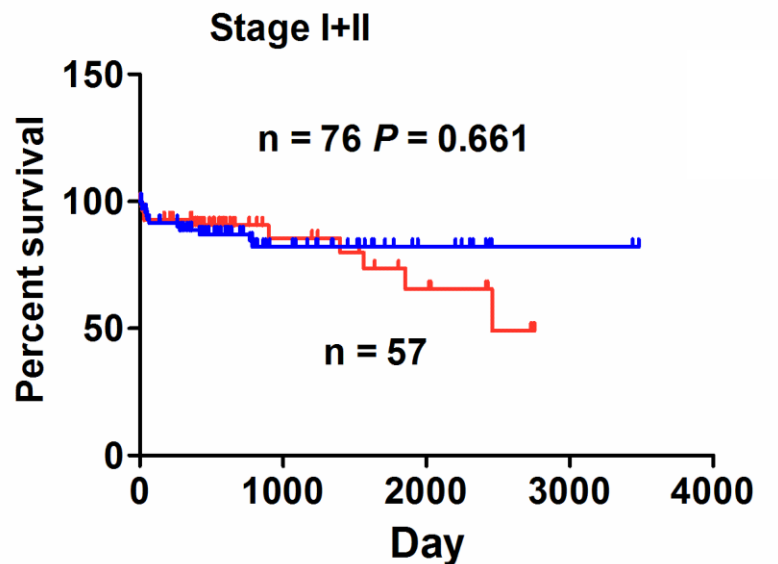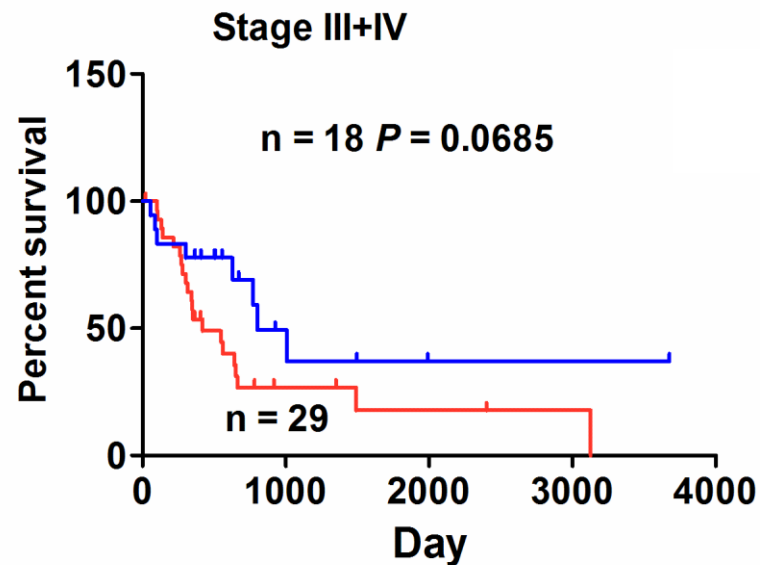**B**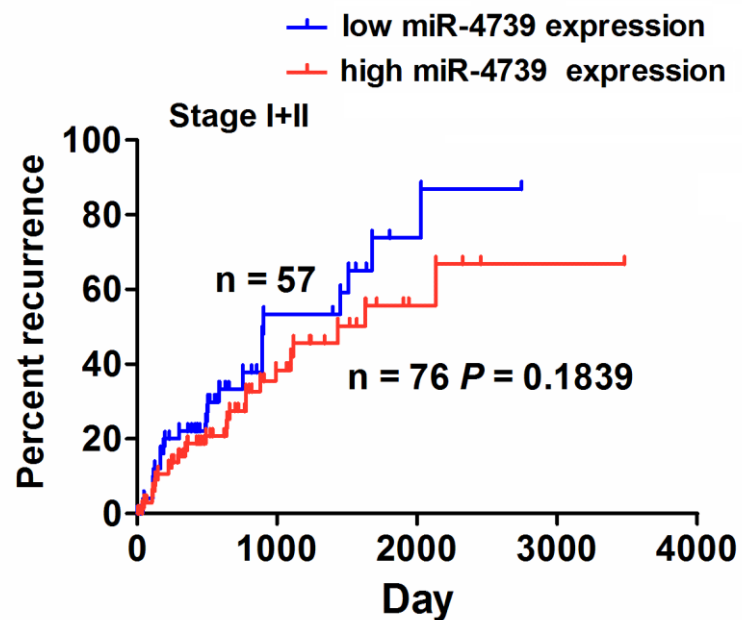

**FIG.S4**

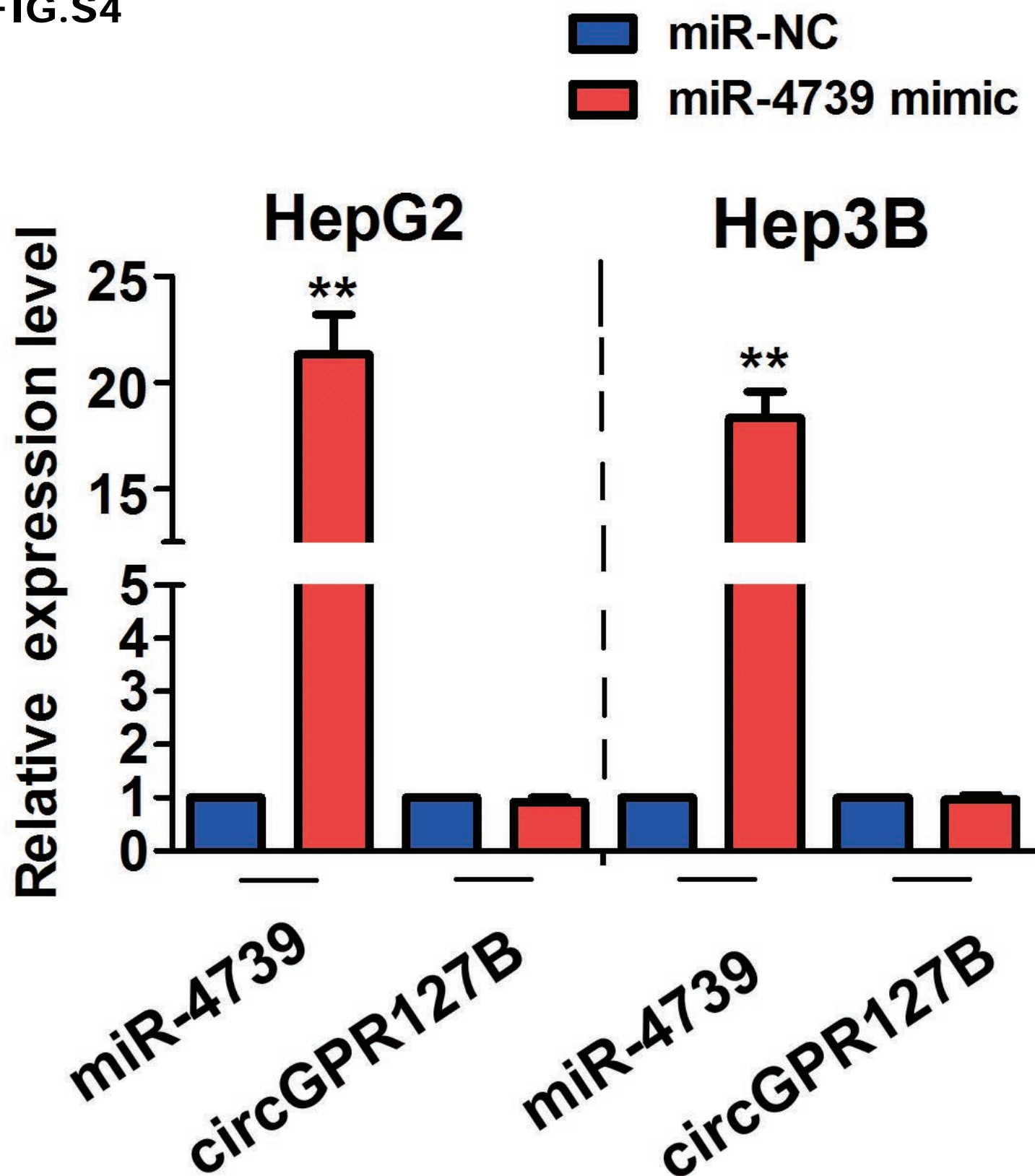

**FIG.S5**

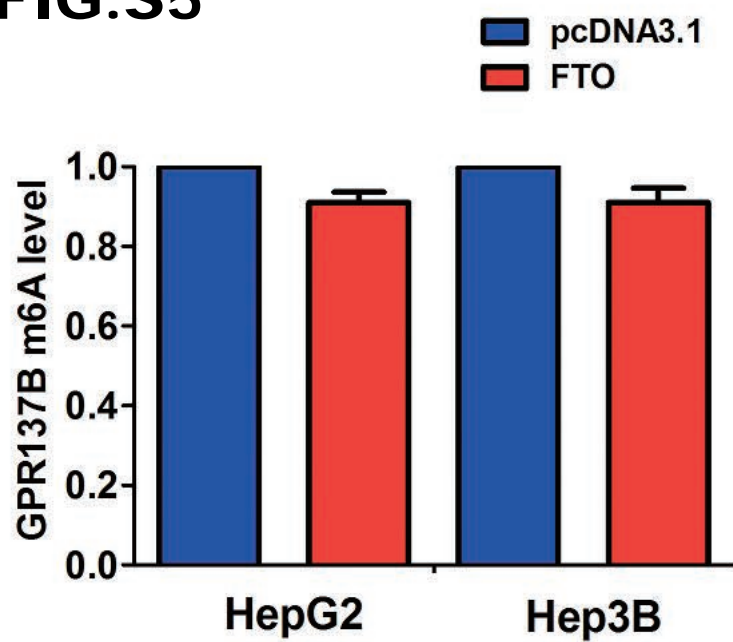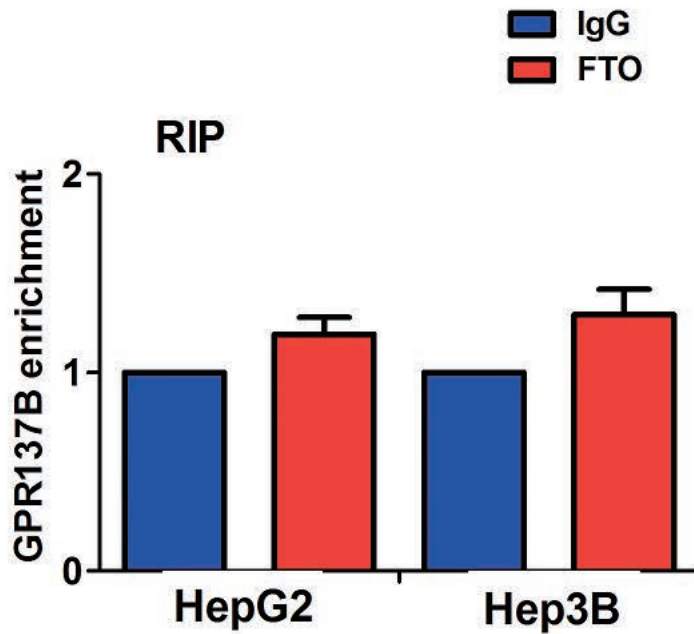

**FIG.S6**

**n = 3** ***P* = 0.4489**

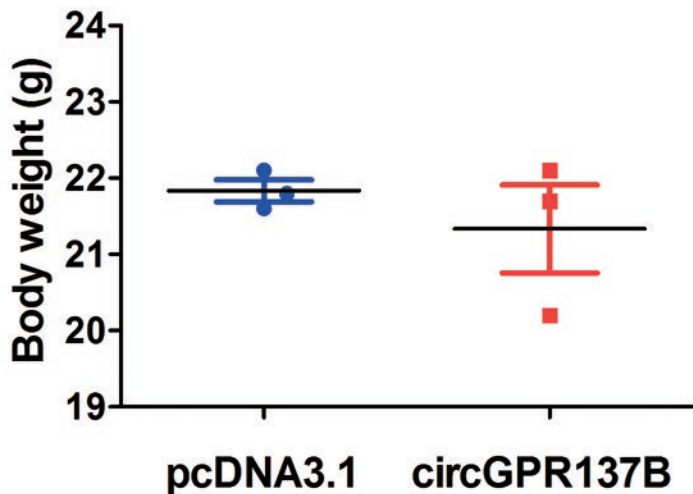

Supplement: Supplementary file 1 — Additional file 1: Supplementary Figure S1. Kaplan–Meier analysis of the association of circGRP137B high or low expression with overall survival in advanced stage cases. Supplementary Figure S2. TCGA analysis of the expression levels of GPR137B and its correlation with miR-4739 expression in HCC tissue samples. Supplementary Figure S3. Kaplan–Meier analysis of the association of miR-4739 high or low expression with (A) overall survival and (B) tumor recurrence in HCC and early/late stage cases. Supplementary Figure S4. qPCR analysis of the effects of miR-4739 mimics on the expression of circGPR137B in HepG2 and Hep3B cell lines. Supplementary Figure S5. MeRIP analysis of the effects of FTO on the m6A levels of GPR137B and RIP analysis of the binding between FTO and GPR137B in HepG2 and Hep3B cell lines. Supplementary Figure S6. Comparison of the body weight between circGPR137B and control groups in liver tumor peritoneal metastasis models. [file 12943_2022_1619_MOESM1_ESM.pdf]
