# Supplementary material for: CircGPR137B/miR-4739/FTO feedback loop suppresses tumorigenesis and metastasis of hepatocellular carcinoma
Source: Mol Cancer. 2022 Jul 20;21:149. doi: 10.1186/s12943-022-01619-4 (PMC9297645; doi:10.1186/s12943-022-01619-4)
Supplement: Supplementary file 2 — Additional file 2: Table S1. The primer sequences. Table S2. Correlation of circGPR137B expression with clinicopathological features of HCC patients. Table S3. Univariate and multivariate Cox regression analysis of the association of circGPR137B with poor survival in HCC patients. Table S4. The correlation of miR-4739 expression with clinicopathologic characteristics of HCC patients. Table S5. Cox regression analysis of miR-4739 expression as survival predictor. Table S6. Cox regression analysis of miR-4739 expression as recurrence predictor. Table S7. RNA-binding protein Ago2 sites matching flanking regions of circGPR137B. Table S8. The correlation of FTO expression with clinicopathologic characteristics of HCC patients. Table S9. Cox regression analysis of FTO expression as survival predictor. [file 12943_2022_1619_MOESM2_ESM.pdf]

Supplementary figures

Supplementary Figure S1. Kaplan–Meier analysis of the association of circGRP137B high or low expression with overall survival in advanced stage cases.

Supplementary Figure S2. TCGA analysis of the expression levels of GPR137B and its correlation with miR-4739 expression in HCC tissue samples.

Supplementary Figure S3. Kaplan–Meier analysis of the association of miR-4739 high or low expression with **(A)** overall survival and **(B)** tumor recurrence in HCC and early/late stage cases.

Supplementary Figure S4. qPCR analysis of the effects of miR-4739 mimics on the expression of circGPR137B in HepG2 and Hep3B cell lines.

Supplementary Figure S5. MeRIP analysis of the effects of FTO on the m^6A levels of GPR137B and RIP analysis of the binding between FTO and GPR137B in HepG2 and Hep3B cell lines.

Supplementary Figure S6. Comparison of the body weight between circGPR137B and control groups in liver tumor peritoneal metastasis models.

Supplementary Tables

Table S1 The primer sequences

**Gene name**	**Primer sequence**	Annealing temperature (℃)	Product length (bp)
β-actin（Human）	F:5' GTGGCCGAGGACTTTGATTG 3'<br>R:5’CCTGTAACAACGCATCTCATATT 3’	60	73
circGPR137B	F:5’ 5'CGTGTGTCAAGTGACTGCCAT3’3’<br>R:5’ ACTTGGCTTTGAAAATCACCTG 3’	60	161
miR-4739	F: 5’AAGGGAGGAGGAGCGGAG 3’<br>R: 5’GTGCGTGTCGTGGAGTCG 3’	60	89
U6	F: GCTTCGGCAGCACATATACTAAAAT<br>R:5’CGCTTCACGAATTTGCGTGTCAT3’	60	63
FTO	F: 5’ ACTTGGCTCCCTTATCTGACC 3’<br>R:5’ TGTGCAGTGTGAGAAAGGCTT3’	60	145
GPR137B	F: 5’ GGTAAAGACGGGAAATTGGGAG 3’<br>R:5’ AGCACGAAGAGCGTGTCATTA3’	60	75

circGPR137B cDNA sequence (Red: primer location; Blue, m6A motif and the underlined A is the potential methylation location).

GTGATTTTCAAAGCCAAGTCAAAATATTCTCCAGAATTACTCAAATACCGGTTGCCCCTCTACCT
GGCCTCCCTCTTCATCAGCCTTGTTTTCCTGTTGGTGAATTTAACCTGTGCTGTGCTGGTAAAGAC
GGGAAATTGGGAGAGGAAGGTTATCGTCTCTGTGCGAGTGGCCATTAATGACACGCTCTTCGTG
CTGTGTGCCGTCTCTCTCTCCATCTGTCTCTACAAAATCTCTAAGATGTCCTTAGCCAACATTTAC
TTGGAGTCCAAGGGCTCCTCCGTGTGTCAAGTGACTGCCATCGGTGTCACCGTGATACTGCTTT
ACACCTCTCGGGCCTGCTACAACCTGTTCATCCTGTCATTTTCTCAGAACAAGAGCGTCCATTCC
TTTGATTATGACTGGTACAATGTATCAGACCAGGCAGATTTGAAGAATCAGCTGGGAGATGCTG
GATACGTATTATTTGGAGTGGTGTTATTTGTTTGGGAACTCTTACCTACCACCTTAGTCGTTTATTT
CTTCCGAGTTAGAAATCCTACAAAGGACCTTACCAACCCTGGAATGGTCCCCAGCCATGGATTC
AGTCCCAGATCTTATTTCTTTGACAACCCTCGAAGATATGACAGTGATGATGACCTTGCCTGGAA
CATTGCCCCTCAGGGACTTCAGGGAGGTTTTGCTCCAGATTACTATGATTGGGGACAACAAACT
AACAGCTTCCTGGCACAAGCAGGAACTTTGCAAGACTCAACTTTGGATCCTGACAAACCAAGCC
TTGGGTAGCATCAGTTAACAGTTTTATGGACGATTCCTCAGATGAAAAGCTTCAGAAAAGCATA
GTGACAGCTGAATTTTTAGGGCACTTTTCCTTAAGAAATAGAACTTGATTTTTATTTGTTACAGGT

TTCCAATGGCCCCATAGGAATAAGCAATAATGTAGACTGATAAACCCTTATTTTAGTACTAAAGA
GGGAGCCTTGCTATTTCAGTGGGTATAATTTAAACTTTTTAAAGAAAATCTGTACTTTTATAAAGA
TGTATTTTGTATAACTTAAATAATAATGCTAAAGTATACTAGGGTTTTTTTTTCTTGAGAATGTTAC
TGCAATCATGTTGTAGTTTGCACAGACTTTTATGCATAATTCACTTTAAAAATATAGAATATATGG
TCTAATAGTTTTTTAAAGCTTTTGGACTAAAGTATTCCACAAATCTTACCTCTTTAGGTCACTGAT
GGTCACTCCGATTCTGAGTGCCACATTGGTAGACTCCTAAAATACAGTTGACAACTTAGCCAATT
GCAACTCCAGTGTTGATAATTAAAATGAAATGGTAAAGCAGCAGACTGTAAGGTCTTTAGAGAT
TTTTTTTTTAAGGTTCAGGCCGTAGGTTCCTCAAGGAATCTCTTAAGTTTTGCCCAAAGACTGGTA
CTTCCTTTCAGTAGGGCGCTAATGTATACACATTAATGATAAGTTGATAACATTAAAAATGTAGC
TGACTTATCCTATTAAACCTCCTCTGCTATGTTCACAGA

Table S2 Correlation of circGPR137B expression with clinicopathological features of HCC patients

parameters	Cases (n)	circGPR137B expression		*P*
		Low	High	
	87	n=29	n=58	
Age				
≥60	21	8	13	
<60	66	21	45	0.597
Gender				
Female	9	2	7	
Male	78	27	51	0.458
Tumor size (cm)				
<3.5	18	7	11	
≥3.5	69	22	47	0.577
Pathological stage				
I+II	58	18	40	
III	29	11	18	0.522
With cirrhosis				
Yes	31	8	23	
No	56	21	35	0.271
TNM staging				
1+2	46	13	33	
3+4	41	16	25	0.291

Table S3 Univariate and multivariate Cox regression analysis of the association of circGPR137B with poor survival in HCC patients

Parameter	Univariate	Multivariate analysis		
	P	*P*	HR	95%CI
Age (≥60 vs. <60 years)	0.522	NA		
Gender (Male vs. Female)	0.491	NA		
Tumor size (≥3.5 vs. <3.5 cm)	0.135	NA		
With cirrhosis (Yes vs. No)	0.969	NA		
Pathological staging (III vs. I/II)	0.283	NA		
TNM staging (3/4 vs. 1/2)	0.006	0.011	2.053	1.183-3.565
circGPR137B expression (High vs. low)	0.008	0.015	0.506	0.292-0.878

NA: not analyzed

Table S4 The correlation of miR-4739 expression with clinicopathologic characteristics of HCC patients

Variables	Cases (n)	miR-4739		*P* value
		High	Low	
Total	180	86	94	
Age (years)				
≥60	91	46	45	
<60	89	40	49	0.460
Gender				
Male	125	56	69	
Female	55	30	25	0.259
Pathological stage				
Ⅰ/Ⅱ	133	57	76	
Ⅲ/Ⅳ	47	29	18	0.029
T stage				
T1/T2	134	57	77	
T3/T4	46	29	17	0.018
N stage				
Negative	133	64	69	
Positive	47	22	25	1.000
M stage				
Negative	144	70	74	
Positive	36	16	20	0.711

Table S5 Cox regression analysis of miR-4739 expression as survival predictor

Variables	Univariate Cox regression analysis		Multivariate Cox regression analysis	
	RR (95% CI)	*P* value	RR (95% CI)	*P* value
Age (years)				
<60 vs. ≥60	1.671 (0.943 to 2.962)	0.079	NA	NA
Gender				
Male vs. Female	0.718 (0.406 to 1.270)	0.255	NA	NA
Pathological stage				
Ⅲ/Ⅳ vs. Ⅰ/Ⅱ	4.596 (2.605 to 8.108)	<0.0001	6.009 (0.783 to 46.135)	0.085
T stage				
T3+T4 vs. T1+T2	4.430 (2.517 to 7.795)	<0.0001	0.707 (0.091 to 5.468)	0.740
N staging				
Positive vs. Negative	0.912 (0.453 to 1.836)	0.797	NA	NA
M stage				
Positive vs. Negative	0.765 (0.342 to 1.709)	0.513	NA	NA
miR-4739 expression				
High VS. Low	1.973 (1.111 to 3.503)	0.020	1.661 (0.921 to 2.997)	0.092

NA: not analyzed

Table S6 Cox regression analysis of miR-4739 expression as recurrence predictor

Variables	Univariate Cox regression analysis		Multivariate Cox regression analysis	
	RR (95% CI)	*P* value	RR (95% CI)	*P* value
Age (years)				
<60 vs. ≥60	1.098 (0.703 to 1.715)	0.681	NA	NA
Gender				
Male vs. Female	1.014 (0.629 to 1.635)	0.954	NA	NA
Pathological stage				
Ⅲ/Ⅳ vs. Ⅰ/Ⅱ	2.777 (1.743 to 4.425)	<0.0001	6.827 (0.909 to 51.261)	0.062
T stage				
T3+T4 vs. T1+T2	2.684 (1.681 to 4.286)	<0.0001	0.380 (0.050 to 2.884)	0.349
N staging				
Positive vs. Negative	0.661 (0.370 to 1.183)	0.163	NA	NA
M stage				
Positive vs. Negative	0.785 (0.432 to 1.426)	0.426	NA	NA
miR-4739 expression				
High VS. Low	1.919 (1.222 to 3.015)	0.005	1.841 (1.164 to 2.910)	0.009

NA: not analyzed

Table S7 RNA-binding protein Ago2 sites matching flanking regions of circGPR137B

circRNA	Tag Name	% Identity	Alignment Length	Tag Start	Tag End	circRNA Start	circRNA ENd
hsa_circ_0017114	HPSD1_503_G666.1_236373055_30	100.00	30	1	30	+829	+858
hsa_circ_0017114	HPSEF_1262_G1500.1_236373120_32	100.00	32	1	32	+901	+932
hsa_circ_0017114	HPSLC_1024_G1227.1_236372244_29	100.00	29	1	29	+32	+60
hsa_circ_0017114	HPSLC_1025_G1228.1_236372345_32	100.00	32	1	32	+122	+153
hsa_circ_0017114	HPSLC_1026_G1229.1_236373055_30	100.00	30	1	30	+829	+858

Table S8 The correlation of FTO expression with clinicopathologic characteristics of HCC patients

Variables	Cases (n)	FTO High	FTO Low	*P* value
Total	338	255	83	
Age (years)				
≥60	181	145	36	
<60	157	110	47	0.042*
Gender				
Male	231	174	57	
Female	107	81	26	1.000
Pathological stage				
Ⅰ/Ⅱ	251	193	58	
Ⅲ/Ⅳ	87	62	25	0.313
T stage				
T1/T2	254	196	58	
T3/T4	84	59	25	0.242
N stage				
Negative	248	182	66	
Positive	90	73	17	0.156
M stage				
Negative	258	188	70	
Positive	80	67	13	0.054

Table S9 Cox regression analysis of FTO expression as survival predictor

Variables	Univariate Cox regression analysis		Multivariate Cox regression analysis	
	RR (95% CI)	*P* value	RR (95% CI)	*P* value
Age (years)				
<60 vs. ≥60	1.292 (0.893 to 1.870)	0.173	NA	NA
Gender				
Male vs. Female	0.781 (0.537 to 1.134)	0.194	NA	NA
Pathological stage				
Ⅲ/Ⅳ vs. Ⅰ/Ⅱ	2.420 (1.671 to 3.503)	<0.001	2.050 (0.280 to 14.988)	0.479
T stage				
T3+T4 vs. T1+T2	2.440 (1.683 to 3.538)	<0.001	1.234 (0.168 to 9.067)	0.837
N staging				
Positive vs. Negative	1.407 (0.936 to 2.115)	0.101	NA	NA
M stage				
Positive vs. Negative	1.598 (1.062 to 2.464)	0.024	1.277 (0.728 to 2.240)	0.395
FTO expression				
High VS. Low	0.624 (0.423 to 0.921)	0.018	0.602 (0.405 to 0.894)	0.012

NA: not analyzed
